# Trafficking and release of *Leishmania* metacyclic HASPB on macrophage invasion

**DOI:** 10.1111/j.1462-5822.2012.01756.x

**Published:** 2012-02-24

**Authors:** Lorna M MacLean, Peter J O'Toole, Meg Stark, Jo Marrison, Claudia Seelenmeyer, Walter Nickel, Deborah F Smith

**Affiliations:** 1 Centre for Immunology and Infection, Department of Biology/Hull York Medical School, University of YorkYork YO10 5DD, UK; 2 Technology Facility, Department of Biology, University of YorkHeslington, York YO10 5DD, UK; 3 Heidelberg University Biochemistry CenterIm Neuenheimer Feld 328, 69120 Heidelberg, Germany

## Abstract

Proteins of the *Leishmania* hydrophilic acylated surface protein B (HASPB) family are only expressed in infective parasites (both extra- and intracellular stages) and, together with the peripheral membrane protein SHERP (small hydrophilic endoplasmic reticulum-associated protein), are essential for parasite differentiation (metacyclogenesis) in the sand fly vector. HASPB is a ‘non-classically’ secreted protein, requiring N-terminal acylation for trafficking to and exposure on the plasma membrane. Here, we use live cell imaging methods to further explore this pathway to the membrane and flagellum. Unlike HASPB trafficking in transfected mammalian cells, we find no evidence for a phosphorylation-regulated recycling pathway in metacyclic parasites. Once at the plasma membrane, HASPB18–GFP (green fluorescent protein) can undergo bidirectional movement within the inner leaflet of the membrane and on the flagellum. Transfer of fluorescent protein between the flagellum and the plasma membrane is compromised, however, suggesting the presence of a diffusion barrier at the base of the *Leishmania* flagellum. Full-length HASPB is released from the metacyclic parasite surface on to macrophages during phagocytosis but while expression is maintained in intracellular amastigotes, HASPB cannot be detected on the external surface in these cells. Thus HASPB may be a dual function protein that is shed by the infective metacyclic but retained internally once *Leishmania* are taken up by macrophages.

## Introduction

Protozoan parasites of the *Leishmania* genus are causative agents of a diverse spectrum of global infectious diseases, the Leishmaniases (reviewed in Murray *et al*., 2005). The *Leishmania* parasite differentiates as it cycles between an intracellular niche within the phagosome of mammalian macrophages and an extracellular niche within its sand fly vector: the immotile amastigote in the host is taken up during vector blood-feeding and transforms into a flagellated promastigote. Completion of the parasite life cycle requires promastigote differentiation into a non-replicative metacyclic cell that is pre-adapted for transmission from vector to host during a subsequent blood meal (Bates, 2007). The metacyclics enter resident dermal macrophages and differentiate into replicative amastigotes for dissemination to other tissues, often inducing inflammatory responses and persistent infection. The fate of these intracellular parasites determines disease type, which can range from cutaneous or mucocutaneous infection to the often fatal visceral leishmaniasis.

The surface of *Leishmania* parasites, the interface between the parasite and its external environment (whether vector or host) is rich in glycophosphatidylinositol (GPI)-anchored macromolecules which vary in composition between extra- and intracellular life cycle stages (reviewed in Ilgoutz and McConville, 2001). Promastigotes in the sand fly vector have an extensive glycocalyx, comprised of complex glycoconjugates such as lipophosphoglycan (LPG) and a small number of glycoproteins, the best characterized of which is GP63 (Joshi *et al*., 2002; Yao *et al*., 2003). Both of these macromolecules are required for parasite survival in the vector, with LPG undergoing extensive modification during parasite metacyclogenesis (Saraiva *et al*., 1995).

In comparison, the intracellular parasite surface is physically less complex but still composed of lipid-anchored glycoconjugates, the glycoinositolphospholipids(GIPLs; Ralton and McConville, 1998), although these have been shown to be non-essential for parasite survival in the mammalian host (Zufferey *et al*., 2003). Few proteins have yet been described that localize to the amastigote plasma membrane with the exception of the amastin glycoprotein family (reviewed in Jackson, 2010) and the hydrophilic acylated surface proteins (HASPs). The *Leishmania*-specific HASPs are stage-regulated and only expressed in infective metacyclics and/or amastigotes, where they show both inter- and intra-specific variation, principally in their characteristic central amino acid repeat regions (Flinn *et al*., 1994; Rangarajan *et al*., 1995; McKean *et al*., 1997; Alce *et al*., 1999; Denny *et al*., 2000; Depledge *et al*., 2010). The HASP proteins are immunogenic: sera from patients with both visceral and cutaneous leishmaniasis recognize recombinant hydrophilic acylated surface protein B (HASPB) with high specificity and sensitivity (Jensen *et al*., 1996; 1999), while the recently identified orthologous HASPs of *Leishmania braziliensis* are also detected by sera taken from Brazilian leishmaniasis patients (Depledge *et al*., 2010). Vaccination with recombinant *Leishmania donovani* HASPB at low dose can induce long-term protection when administered in the absence of exogenous antigen in mice (Stager *et al*., 2000; 2003), while recombinant HASPB is also immunogenic in dogs and induces significant protection against canine leishmaniasis (Moreno *et al*., 2007).

The HASPs can be categorized with other ‘non-classically’ secreted proteins (including mammalian growth factors and galectins) that lack a conventional signal peptide and reach the cell surface by a route that avoids the classical ER-Golgi secretory pathway (Nickel and Rabouille, 2009). In the case of HASPB, this requires dual acylation at the N-terminus to facilitate trafficking to the plasma membrane (Denny *et al*., 2000). A combination of fluorescence microscopy of fixed parasites, pH-dependent fluorescence assay and biochemical labelling and fractionation were used to show that the first 18 amino acids of *Leishmania major* HASPB are sufficient to target GFP to the promastigote plasma membrane where it can be exposed to the external environment (Denny *et al*., 2000). A similar trafficking pathway is present in mammalian cells (Denny *et al*., 2000; Stegmayer *et al*., 2005). N-terminal myristoylation and palmitoylation are essential modifications that facilitate this process: while the wild-type HASPB18–GFP reporter protein is transported to the plasma membrane, disruption of the N-myristoylation site (by a G2A mutation) prevents both acylation events, resulting in retention of the HASPB18–GFP G2A reporter protein in the cytosol (Denny *et al*., 2000). In contrast, disruption of the N-terminal palmitoylation site (by a C3S mutation) leads to concentration of the HASPB18–GFP C3S reporter protein in the vicinity of the Golgi (Denny *et al*., 2000), a location consistent with trafficking via an exocytic pathway to the surface. More recently, a role for reversible phosphorylation as a molecular switch for plasma membrane targeting of acylated SH4 domain proteins has been proposed (Tournaviti *et al*., 2009), using the N-terminus of *L. major* HASPB as an example, in fusion with GFP in mammalian cells. In this study, identification of an N-terminal threonine as a substrate for phosphorylation led to discovery of a phosphorylation/dephosphorylation cycle that can recycle SH4 domain proteins between the plasma membrane and perinuclear membranes via endosomal transport. Whether a similar mechanism occurs in the *Leishmania* parasite was not explored in this analysis.

At the plasma membrane, surface exposure of the HASPB–GFP reporter protein has been detected biochemically by surface biotinylation (Denny *et al*., 2000). In parallel, wild-type HASPB has been localized to sphingolipid/sterol-rich microdomains in metacyclic stages of *L. major* (Denny *et al*., 2001; Denny and Smith, 2004), a localization that correlates with structures visualized at the metacyclic plasma membrane by fracture-flip electron microscopy (Pimenta *et al*., 1994; Sacks *et al*., 1995). These observations suggest that HASPB function is required at the parasite surface either during parasite transmission to the host or during and after establishment of parasites within host macrophages. The functions of these unusual proteins have been investigated in null transgenic *L. major* parasites generated by targeted deletion of a ∼ 12 kb region of chromosome 23 (the *L. major* cDNA16 locus; Flinn and Smith, 1992) that contains the HASP and small hydrophilic endoplasmic reticulum-associated protein (SHERP) genes. These null mutants do not show a strong phenotype in cultured macrophages or susceptible BALB/c mice as compared with wild-type parasites (McKean *et al*., 2001). However, recent observations have revealed an essential role for products of the LmcDNA16 locus in metacyclogenesis in the sand fly vector, suggesting that the HASP and/or SHERP gene products are crucial for parasite transmission *in vivo* (Sadlova *et al*., 2010). These new data renew our interest in the function of HASPB at the plasma membrane in both metacyclic and amastigote parasites. Here, we use live and fixed cell imaging techniques to further investigate the route by which HASPB is transported to and exposed on the plasma membrane and show that this protein is released from the metacyclic parasite surface during phagocytosis by macrophages. We also use fluorescence recovery after photobleaching (FRAP) to analyse the dynamics of HASPB in live cells, leading to the hypothesis that *Leishmania* parasites have some form of diffusion barrier that separates the cell body membrane and flagellum, a mechanism that might regulate trafficking to and from the flagellum.

## Results

### Transport of *L. major* HASPB to the parasite plasma membrane

**Fig 1:**
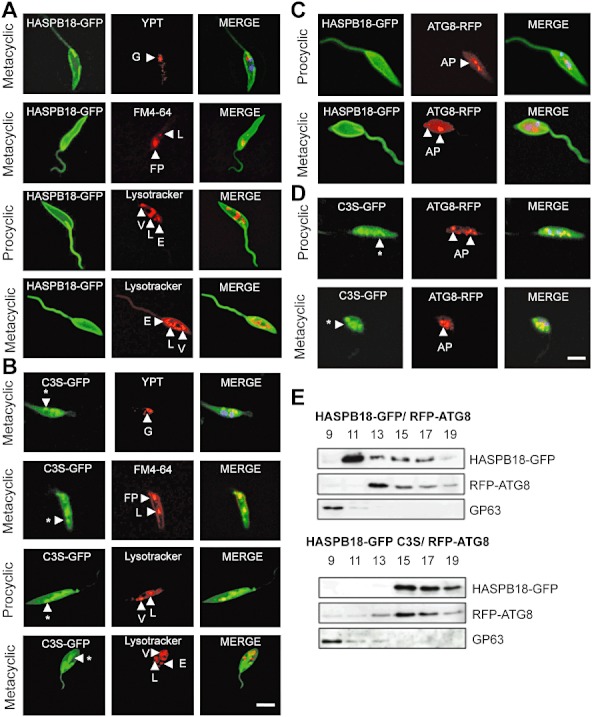
HASPB18–GFP localization in *L. major* promastigotes. Both fixed and live cell imaging methods were used to localize the HASPB18–GFP (A) and HASPB18–GFP C3S fusion proteins (B) in transfected *L. major* parasites [all parasites analysed were early passage cells (≤ p6)]. A and B. Top panel: Metacyclic parasites expressing HASPB18–GFP or HASPB18–GFP C3S were fixed and permeabilized, prior to labelling with rabbit anti-YPT and subsequent detection using AlexaFluor-647-conjugated goat anti-rabbit IgG. Lower three panels: Live metacyclics or procyclics expressing either GFP fusion protein were imaged after immobilization in PBS-primed CyGEL and labelling with Lysotracker RED DND-99 or FM4-64 for 90 min. *Vesicular structures of unknown origin. C and D. Live cell staining of HASPB18–GFP (C) and C3S (D) parasites that are also expressing the autophagosomal marker, RFP-ATG8. E. Density gradient fractionation of cell lysates from metacyclic parasites of the HASPB18–GFP/RFP-ATG8 lines analysed in (C) and (D), immunoblotted with anti-GFP, anti-RFP or anti-GP63. Arrowheads indicate subcellular compartments as follows: G, Golgi; L, lysosome; FP, flagellar pocket; E, endosome; V, vesicle; AP, autophagosome. Size bar, 5 µm (all images at the same magnification).

In this study, we have focused on the extracellular stage in the parasite life cycle in which HASPB is expressed *in vivo*, the flagellated infective metacyclic cell. Fluorescence microscopy of fixed and live parasites episomally overexpressing fluorescent reporter proteins has been used to compare the localization of the HASPB18–GFP and HASPB18–GFP C3S fusion proteins in metacyclic (from stationary phase, Day 7 culture) as compared with procyclic (from logarithmic phase, Day 2 culture) cells (Fig. 1). These lines are described in Table S1A. In the smaller-bodied metacyclics, expression of HASPB18–GFP is clearly visible in fixed cells at the plasma membrane and flagellum (Fig. 1A), as shown previously in transfected procyclics (Denny *et al*., 2000). Antibodies specific for the *L. major* Golgi-associated YPT (yeast protein transport) GTPase orthologue (Cappai *et al*., 1993) identify the single Golgi; the HASPB18–GFP C3S fusion protein lacking the cysteine residue required for palmitoylation (Fig. 1B top panel) colocalizes with this organelle, as suggested previously using Bodipy-ceramide (Denny *et al*., 2000). This non-palmitoylated fusion protein, while excluded from the flagellum, is also observed in other subcellular vesicles, however, and these do not colocalize with the acidocalcisome marker, vacuolar proton-pyrophosphatase (V-H+-PPase; data not shown).

To analyse this additional protein distribution further, procyclic and metacyclic parasites from the same lines were incubated live with either Lysotracker RED or FM4-64 fluorescent tracers and immobilized in CyGEL for imaging (Fig. 1A and B lower three panels). This method has been used to monitor uptake of FM4-64 over a 90 min time-course, during which time the dye moves from the cell body membrane and flagellar pocket to the lysosome via the endosomal system (Besteiro *et al*., 2006; Price *et al*., 2010). As expected, HASPB18–GFP again showed plasma membrane and flagellar localization in both parasite stages, with Lysotracker RED staining the acidic compartments and FM4-64 found in the flagellar pocket/early endosomes and lysosome under the labelling conditions used. HASPB18–GFP showed some colocalization with FM4-64 in the pocket as expected but was excluded from the acidic vesicles. This localization pattern was also observed in Lysotracker RED-treated immobilized *L. major* expressing the full-length HASPB–GFP fusion protein rather than the N-terminal 18-residue fusion (Fig. S1A), suggesting that exclusion from the acidic vesicles requires only the dual acylated N-terminus of the protein. In contrast, HASPB18–GFP C3S colocalized not only with the Golgi (as shown previously) and lysosome but also with a subset of acidic vesicles that extended along the length of the parasite cell body and were more abundant in metacyclic cells (Fig. 1B lower two panels). These vesicles were rarely observed in permeabilized and late passage cells (≥p10; data not shown).

A population of acidic vesicles has previously been identified in metacyclic *Leishmania* which result from macroautophagy, recently shown to be an essential requirement for metacyclogenesis and parasite virulence (Besteiro *et al*., 2006; 2007). During parasite differentiation, the multivesicular body (MVB)-like network found in multiplicative procyclic parasites matures into a lysosomal-like structure of high lytic capacity and low pH in metacyclic parasites (Besteiro *et al*., 2006), correlating with the appearance of autophagosomes expressing autophagy-related gene 8 (ATG8), the ubiquitin-like protein required for vesicle formation in yeast (reviewed in Kiel, 2010). To investigate whether the additional vesicles visualized with HASPB18–GFP C3S were autophagosomes, HASPB–GFP parasites were transfected with RFP-ATG8, an N-terminal red fluorescence protein reporter in fusion with the autophagic marker protein, ATG8 (Besteiro *et al*., 2006; Williams *et al*., 2006). In Fig. 1D, early passage *L. major* showed partial colocalization of GFP- and RFP-fluorescent vesicles in HASPB18–GFP C3S parasites, in contrast to the lack of colocalization observed in HASPB18–GFP cells (Fig. 1C), suggesting that a proportion of the non-palmitoylated reporter protein is associated with this compartment. This observation correlated with changes in the subcellular fractionation of markers, analysing lysates from both cell lines separated by density gradient centrifugation prior to immunoblotting (Fig. 1E). As expected, most of the HASPB18–GFP was found in the plasma membrane fractions (9–11), colocalizing with the surface glycoprotein, GP63 (Yao *et al*., 2003), although the acylated HASPB separates distinctly from the GPI-anchored GP63, suggesting that these may occupy different plasma membrane compartments (as previously proposed: Denny and Smith, 2004). In contrast, loss of palmitoylation resulted in redistribution of the HASPB18–GFP C3S fusion protein within the gradient, colocalizing predominantly with RFP-ATG8 in these cells (although there is some shift in RFP-ATG8 fractionation as compared with the fully acylated cell extract, an observation of unknown functional significance). These data suggest that HASPB and the autophagosomal marker, ATG8, co-segregate when HASPB is myristoylated but not palmitoylated, potentially identifying the additional vesicles observed in Fig. 1B and D as autophagosomes. One interpretation of these results is that, while the HASPB18–GFP reporter protein is transported rapidly to the inner leaflet of the plasma membrane following dual acylation, the non-palmitoylated protein is retained at the Golgi (as previously shown in fixed cells) but saturates that site and is subsequently compartmentalized into autophagosomes prior to destruction. It is interesting to note that the GFP quenching commonly observed at pH < 5.5 (Kneen *et al*., 1998) is not evident in these acidic vesicles, suggesting either that their pH is at least 5.5 or that the HASPB18–GFPC3S protein is localized on the external vesicular surface.

### Is phosphorylation of the HASPB N-terminus required for transport to the plasma membrane in metacyclic *L. major*?

**Fig 2:**
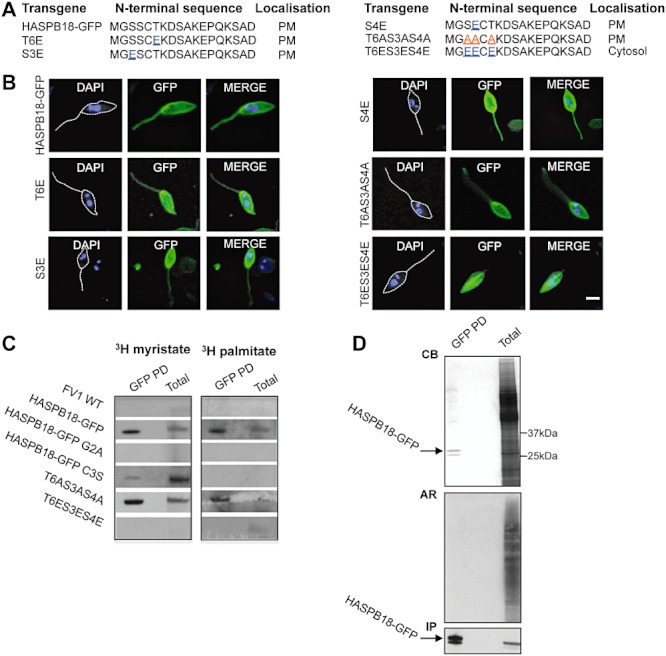
Analysis of acylation and phosphorylation at the HASPB N-terminus. A. HASPB18–GFP reporter constructs used to investigate protein localization in (B) and acylation in (C). The N-terminal 18 residues contained in HASPB18–GFP are shown at the top; mutations in downstream residues are underlined in the sequences below; the localization of the GFP products at the plasma membrane (PM) or in the cytosol in (B) is indicated. B. Localization of HASPB18–GFP in metacyclic *L. major* parasites carrying the mutated reporter constructs shown in (A), as determined by imaging by confocal microscopy. Size bar 5 µM, dashed lines represent the outline of the cell derived from the DIC image (not shown). C. Determination of the acylation status of the GFP products analysed in (A) and (B) by radiolabelling with ^3^H-myristate (left hand panel) or ^3^H-palmitate (right hand panel), followed by SDS-PAGE analysis of total parasite lysates (Total) and products immunoprecipitated from these lysates with anti-GFP (GFP PD). The Coomassie blue-stained gels from this analysis, imaged prior to autoradiography, are shown in Fig. S2. D. Determination of the phosphorylation status of HASPB18–GFP in metacyclic parasites by ^32^P-orthophosphate labelling. Analysis of total parasite lysate and products immunoprecipitated with anti-GFP was carried out by SDS-PAGE and Coomassie blue staining (CB) followed by autoradiography (AR). Samples were also immunoblotted with anti-GFP to confirm the presence and integrity of HASPB18–GFP (IP).

To further delineate the mechanism required for movement of dual acylated HASPB from the Golgi to the plasma membrane, and address whether a phosphorylation/dephosphorylation cycle is important in this process, we investigated the phosphorylation status of the HASPB N-terminus in metacyclic late passage *L. major*. Starting with the HASPB18–GFP reporter construct used above, we first made a series of mutations in the N-terminal domain (primers listed in Table S2), corresponding to those investigated previously (Tournaviti *et al*., 2009). These sequentially remove potential sites for modification by phosphorylation (Fig. 2A). The localization of the reporter proteins expressed from these constructs, following transfection into *L. major*, was observed by fluorescence microscopy of metacyclic parasites. As shown in Fig. 2B, the wild-type reporter protein together with the T6E, S3E, S4E and T6ES3ES4E reporters all showed the characteristic plasma membrane and flagellar localization observed with full-length HASPB18–GFP (Fig. 1A); only the T6ES3ES4E mutant protein was mislocalized to the cytosol. These data suggest that, while single mutations have no effect, removal of all three potential sites of phosphorylation in the N-terminus of HASPB does affect reporter localization. However, the experiments do not address the acylation status of these mutant proteins. This property was investigated by radiolabelling with ^3^H-myristate and ^3^H-palmitate and comparison of total labelled products with those immunoprecipitated with anti-GFP antibody (Fig. 2C). As expected, there was no detectable signal in wild-type *L. major* parasites while the HASPB18–GFP reporter protein was both N-myristoylated and palmitoylated, as was the T6AS3AS4A reporter; both of these localize to the parasite plasma membrane. Also as expected, the HASPB18–GFP C3S reporter was N-myristoylated but not palmitoylated while the HASPB18–GFP G2A reporter was not acylated at all under these conditions, as shown previously (Denny *et al*., 2000). In contrast, the cytosolically localized T6ES3ES4E reporter was neither N-myristoylated or palmitoylated, demonstrating that this triple mutation that destroys three potential N-terminal phosphorylation sites also disrupts the acylation site required for modification at the N-terminus. This observation differs from that seen in mammalian cells with the same reporter construct, which was dually acylated under the same assay conditions. We conclude that the T6ES3ES4E mutation disrupts the N-terminal myristoylation motif required to be a substrate of the *Leishmania N*-myristoyl transferase (NMT), confirming the previously observed divergence in NMT recognition motifs between eukaryotic species (Maurer-Stroh *et al*., 2002). In addition, these data question the role of phosphorylation in the mechanism of HASPB trafficking in the parasite as compared with mammalian cells, in which phosphorylation regulates endosomal recycling between the Golgi and the plasma membrane.

To address this latter issue further, we carried out ^32^P-orthophosphate *in vivo* radiolabelling of *L. major* HASPB18–GFP parasites and analysed total parasite lysate post-labelling together with products immunoprecipitated with anti-GFP. As shown in Fig. 2D and Fig. S2, immunoprecipitation successfully pulled down product with the same mobility as HASPB18–GFP that was detectable by Coomassie blue staining (CB); the identity of this product was confirmed by immunoblotting (IB) with anti-GFP (although it is of interest that two bands were immunoprecipitated with anti-GFP rather than the single band detected in total lysate, suggesting the presence of an alternatively processed product or one with altered mobility, or possibly a degradation product). After 24 h exposure to autoradiography (AR), however, no radiolabelled HASPB product was detected, although multiple phosphorylated proteins were detected in the total parasite lysate. Collectively, these data provide no evidence that *L. major* HASPB is phosphorylated at its N-terminus in the metacyclic parasite, although we cannot exclude the possibility that phosphorylation is a transient event which is not detectable by the methods used here. Taken together with the acylation analysis above, however, we conclude that mislocalization of the T6ES3ES4E reporter to the cytosol is due to a lack of N-terminal acylation rather than deletion of a critical phosphorylation site. In this respect, trafficking of HASPB18–GFP reporter proteins may be dissimilar when comparing mammalian cells (Tournaviti *et al*., 2009) with parasites, at least in their endosomal recycling mechanisms.

### HASPB dynamics at the plasma membrane

**Fig 3:**
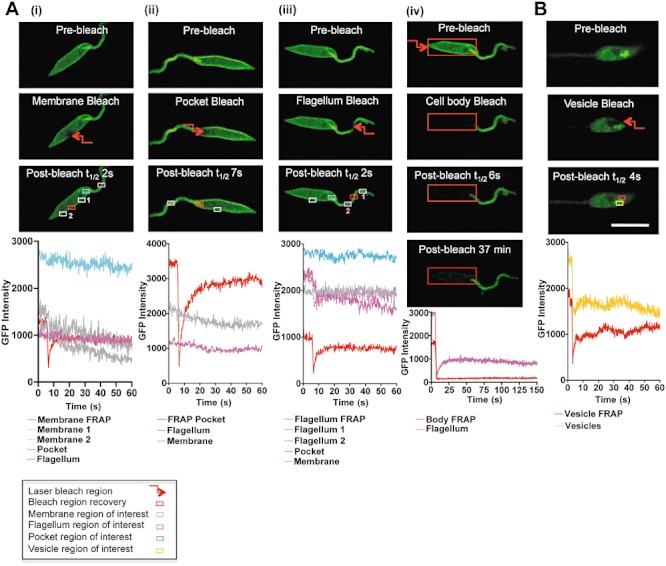
FRAP analysis of HASPB18–GFP intracellular migration. The dynamics and direction of GFP fusion protein movement in live CyGEL-immobilized HASPB18–GFP (Ai–iv) and HASPB18–GFP C3S (B) *L. major* parasites were investigated by FRAP (fluorescence recovery after photobleaching) analysis. Pre-bleach, bleach and post-bleach images are shown of a typical FRAP; the bleached region of interest (ROI) and recovery at other parts of the cell over time are graphically represented below each set of images, plotting GFP intensity versus time. A(i). FRAP (red ROI) at the plasma membrane and analysis of two adjacent plasma membrane ROIs (grey), flagellar pocket ROI (cyan) and flagellum ROI (pink). A(ii). FRAP (red ROI) at the flagellar pocket and simultaneous analysis of plasma membrane ROI (grey) and flagellar pocket ROI (pink). A(iii). FRAP (red ROI) at the flagellum and analysis of two flagellar ROIs (pink), flagellar pocket ROI (cyan) and plasma membrane ROI (grey). A(iv). FRAP (red ROI) of the whole cell body and analysis of this ROI up to 37 min post bleach. B. FRAP (red ROI) of vesicles in metacyclic HASPB18–GFP C3S and analysis of the adjacent vesicle ROI (yellow). Size bar, 10 µm (all images presented at the same magnification).

To investigate the cellular distribution of dual-acylated *L. major* HASPB18–GFP anchored at the plasma membrane (which is detectable on the metacyclic parasite body and flagellum and can be exposed on the outer surface), we first utilized metacyclic early passage wild-type and non-palmitoylated fluorescent reporter lines to examine lateral diffusion in the membrane by FRAP (fluorescence recovery after photo-bleaching) in live cells. Using a laser to rapidly photobleach a small region of interest (ROI) within the HASPB18–GFP fluorescence, the ROI is repopulated by mobile unbleached HASPB18–GFP molecules as the bleached molecules diffuse away until equilibrium is reached. Thus the rate of mobility and percentage recovery can be analysed, giving an indication of the speed of movement and the percentage of mobile versus immobile molecules. Furthermore, by recording the corresponding fluorescence loss induced by photobleaching (FLIP) in another ROI outside of the bleached ROI, the exchange of bleached for unbleached HASPB18–GFP in the cell will allow analysis of the direction of movement of HASPB18–GFP. In Fig. 3A, representative images from a series of experiments measuring FRAP of fluorescent HASPB18–GFP in different regions of the parasite are shown, comparing fluorescence pre- and post-bleaching of signal at the flagellar pocket, cell body membrane, flagellum and whole cell body giving the recovery rates of these individual FRAP experiments (Fig. 3, FRAP data can be viewed as real-time movies in Movies S1–S5; the mean recovery rates for FRAP replicates are graphically represented in Fig. S1B).

FRAP at the cell body membrane (Fig. 3Ai) resulted in a slower HASPB18–GFP recovery rate (*t*_1/2_ mean 6.4 ± 4.8 s) than that observed in the cytosol of HASPB18–GFP C3S (Fig. S1B), due to the diffusion of free cytosolic GFP (*t*_1/2_ mean 1.1 ± 0.3 s), observations consistent with membrane-association of HASPB18–GFP (as shown by immunoblotting subcellular fractions; Fig. 1E). Cell body membrane FRAP correlated with a high percentage FLIP at the cell body membrane ROI (grey) on both sides of the bleached region, and to a lesser extent within the pocket region (cyan), but no loss of fluorescence was observed within the flagellum (pink) (Fig. 3Ai, lower graph). These data support a model in which HASPB18–GFP can undergo bidirectional movement within the lipid bilayer at the cell body membrane and movement to the flagellar pocket, but movement from the cell body membrane to the flagellum is restricted.

FRAP at the flagellar pocket (Fig. 3Aii), the sole site of exo- and endocytosis in the parasite, showed relatively slow HASPB18–GFP diffusion (mean *t*_1/2_ 9.9 ± 3.9 s) as compared with other membrane areas, which may result from the complex structure of the pocket due to membrane invagination. Flagellar pocket FRAP correlated with FLIP recorded at the cell body membrane ROI (grey), with only a marginal effect at the flagellum ROI (pink) over the same time-course, as shown in the graph below. It is of note that the flagellar pocket exhibited brighter fluorescence than the other membranous regions sampled [compare flagellar pocket levels in red (plot Aii) and cyan (plots Ai and Aiii) with cell body membrane levels (grey) and flagellum levels (pink)]. This may be due to a higher concentration of protein in the pocket region correlating with the multiple membrane bilayers present; bleaching through these layers thus results in more molecules being affected while also creating a longer diffusion path and hence, a slower recovery rate. This interpretation is supported by the loss of fluorescence signal outside the bleached area in the membrane over time (Fig. 3Aii, grey plot), indicative of HASPB18–GFP movement from the cell body membrane to the flagellar pocket. The loss of only a small percentage of fluorescence from the flagellum during the same period most likely reflects redistribution from that portion of the flagellum located within the pocket itself rather than movement from the main flagellar structure to the pocket.

FRAP at the flagellum alone (Fig. 3Aiii) gave a similar recovery time (flagellum mean *t*_1/2_ 4.6 ± 2.7 s; Fig. S1B) as at the cell body membrane, suggesting that HASPB18–GFP also moves freely within the lipid bilayer at the flagellum. Flagellum FRAP led to fluorescence recovery and a high percentage FLIP at the flagellum on both sides of the bleach region, indicative of bidirectional HASPB–GFP movement on the flagellum. In contrast, no FLIP was observed between the pocket or cell body membrane as would be expected if these are distinct compartments and there is no redistribution of protein between them over the timescale measured (results shown up to 60 s but no change was observed up to 156 s; data not shown). Within the flagellum, no preference in direction of movement was recorded, with recovery occurring equally fast either towards or away from the pocket.

FRAP over the main cell body, including the small portion of flagellum that inserts into the flagellar pocket (Fig. 3Aiv), showed that the flagellum had recovered its own fluorescence within the pocket region after 6 s, while the plasma membrane did not recover fluorescence up to 37 min post photo-bleaching. There was no detectable transfer of protein between the flagellum and the flagellar pocket or cell body membrane over this timescale. These results suggest that while dual acylated proteins access the flagellum rapidly in live metacyclic *Leishmania*, they recycle to the parasite body slowly if at all, perhaps indicative of a physical barrier between the cell body membrane and flagellum that restricts protein movement between the flagellum and the cell body under these conditions.

Photobleached vesicles in HASPB18–GFP C3S *L. major* (Fig. 3B) exhibited recovery rates similar to membrane regions (*t*_1/2_ 5.4 ± 1.5 s), supporting the proposed membrane association of the fusion protein in these vesicles. However, the recovery rates appear surprisingly fast for a non-continual complex. This could be in part due to XY shifted out of focus regions of the same complex not being bleached, but being able to repopulate the bleached vesicular region. Indeed, all of the bleached region showed partial recovery as a significant proportion of molecules were bleached in the FRAP process suggesting that these vesicles may be interconnected as one continual body, perhaps the lysosome-like structure characteristic of metacyclic cells (described above).

### HASPB18–GFP is exposed on the plasma membrane in metacyclic *L. major*

Surface exposure of HASPB18–GFP at the plasma membrane has been shown previously by biotinylation (Denny *et al*., 2000). To confirm these observations using whole parasites, cell sorting and imaging methods were employed. The reporter parasite lines described above were grown to late log phase and first labelled as live non-permeabilized parasites with sulfo-succinimidyl-7-amino-4-methylcoumarin-3-acetic acid (Sulfo-NHS-AMCA) (Depledge *et al*., 2010), followed by live staining with anti-GFP for detection of GFP exposure on the cell surface using an AlexaFluor-647-conjugated secondary antibody. Antibody labelling was carried out at 20°C for 30 min (rather than at 37°C for 60 min (Pimenta *et al*., 1994), conditions chosen to minimize antibody capping but maximize signal (Depledge *et al*., 2010).

**Fig 4:**
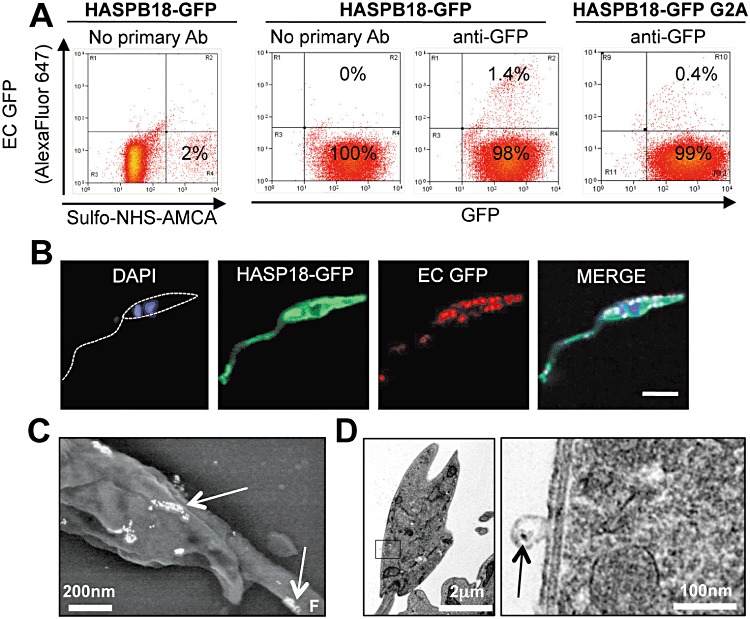
HASPB18–GFP is exposed on the surface of live metacyclic *L. major*. A. Surface HASPB18–GFP exposure was determined by FACS analysis of live HASPB18–GFP *L. major* (following labelling with Sulfo-NHS-AMCA to distinguish between live and dead cells) using mouse anti-GFP and detection by AlexaFluor-647-conjugated goat anti-mouse IgG. Non-N-myristoylated HASPB18–GFP G2A parasites were used as the control for non-surface exposure. The live/dead analysis (left hand panel) detected 2% dead cells in the HASPB18–GFP parasite population; the remaining analyses (centre and right hand panels) are gated on live cells only. EC GFP, extracellular GFP. B. Confocal microscopic analysis of a metacyclic HASPB18–GFP *L. major* labelled using the protocol in (A); extracellular HASPB18–GFP, detected by mouse anti-GFP, decorating the surface of the cell body and flagellum in a punctate distribution, while intracellular protein was detected by GFP fluorescence. Size bar, 5 µm. C. Scanning immunoelectron microscopy of a metacyclic HASPB18–GFP *L. major* incubated live with mouse anti-GFP, prior to fixation in 4% paraformaldehyde and incubation with goat anti-mouse IgG 10 nm gold. Samples were post-fixed in 2.5% glutaraldehyde, dehydrated and carbon coated, prior to visualization. White arrows indicate surface-localized gold labelling, F indicates flagellum protruding from parasite body. D. Transmission immunoelectron microscopy of HASPB18–GFP *L. major* parasites, labelled as in (C). Samples were post-fixed in 2.5% glutaraldehyde/4% formaldehyde and resin embedded, prior to sectioning and visualization. The black box within the whole-cell image represents the area enlarged on the right. Black arrow indicates gold labelling on a surface vesicle.

The live/dead Sulfo-NHS-AMCA assay detected 2% of the total population as dead parasites and these were removed from further fluorescence-activated cell sorting (FACS) analysis (Fig. 4A). Using this method, extracellular (EC) GFP was detected in only ∼ 1% of HASPB18–GFP parasites, with control samples (lacking primary antibody staining or expressing the non-myristoylated HASPB18–GFP G2A reporter protein described in Table S1A) expressing background levels of up to 0.4% EC GFP (Fig. 4A). Cell fractionation and anti-GFP immunoblots confirmed that the HASPB18–GFP G2A protein was not associated with the plasma membrane as previously demonstrated (Denny *et al*., 2000; Fig. S1C). Using confocal microscopy, cells displaying surface HASPB18–GFP, although rare, showed extracellular GFP accumulating in a punctuate pattern on the parasite surface (Fig. 4B) while permeabilized parasites of the same line showed more uniform expression throughout the parasite body and flagellum (data not shown). In these experiments, parasite morphology correlated with the pre-metacyclic stage in culture, characterized by closer proximity of nucleus and kinetoplast than observed in procyclic parasites, shorter body length and relatively longer flagellum. Surface HASPB18–GFP was not detected in HASPB18–GFP G2A (Fig. S1D).

Scanning and transmission immunoelectron microscopy (SIEM and TIEM respectively) were also used to visualize extracellular GFP on HASPB18–GFP parasites. Using anti-GFP and a 10 nm gold-conjugated secondary antibody, GFP was detected in clusters on the external surface of cell body and flagellum by SIEM (Fig. 4C, enlarged version Fig. S5A). This high-resolution pattern is similar to the punctuate staining seen by confocal microscopy and unequivocally confirms that HASPB18–GFP can be detected on the surface of live *Leishmania* promastigotes. Under the conditions used, we cannot discount some antibody capping that would result in a clumping pattern similar to that observed, but this only serves to emphasis the surface exposure of this target antigen.

**Fig 5:**
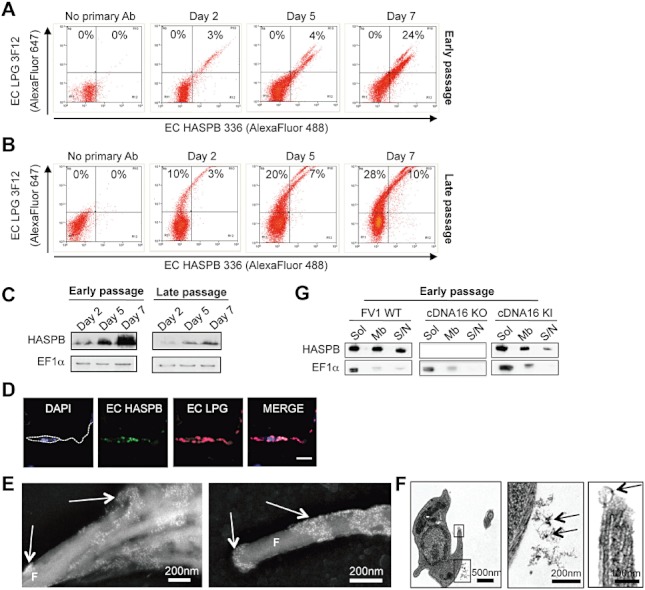
Full-length HASPB is exposed on the surface of metacyclic *L. major*. A and B. Flow cytometry analysis of early passage (p = 3) and late passage (p > 10) *L. major* FVI wild-type parasites sampled from early log phase (Day 2), late log phase (Day 5) and stationary, metacyclic-rich phase (Day 7). Sulfo-NHS-AMCA staining was used to distinguish live/dead cells to allow gating on live parasites only, as in Fig. 4. Live parasites were labelled for detection of EC HASPB (using polyclonal antibody 336 and AlexaFluor-488-conjugated secondary antibody) and metacyclic-specific LPG (EC LPG 3F12, using monoclonal 3F12 antibody and AlexaFluor-647-conjugated secondary antibody). C. Parasites from the same early and late passage cultures were lysed and analysed for HASPB expression by immunoblotting, using EF1-α as a control for equivalent loading. HASPB migrates as a ∼ 36 kDa band under these conditions while EF1-α recognizes a 50 kDa band as the major protein. D. Confocal microscopy of live anti-HASPB- and 3F12-labelled early passage *L. major* (as described in A and B) confirmed surface exposure of full-length HASPB across the entire cell body and flagellum in a punctate pattern. The DAPI-stained kinetoplast and nucleus are visible and their relative positions identify this cell as a metacyclic parasite; this identification is also verified by staining with the metacyclic-specific antibody, 3F12. Extracellular (EC) HASPB colocalized with EC metacyclic LPG in this parasite, which was intact at the time of labelling as indicated by the absence of Sulfo-NHS-AMCA staining. Size bar, 5 µm. E and F. (E) Scanning immunoelectron microscopy and (F) transmission immunoelectron microscopy of live anti-HASPB-labelled early passage stationary-phase *L. major* detected with goat anti-rabbit IgG 10 nm gold. White arrows indicate gold particles in SEM image. The black box within the whole-cell image TIEM represents the area enlarged on the right. Black arrows indicate labelled surface vesicles. F, flagellum. G. HASPB expression and shedding by early passage wild-type *L. major* and LmcDNA16 mutant metacyclic parasites either null for (K0) or re-expressing (KI) the LmcDNA16 locus. Cell fractionation of Day 7 cultures was used to generate membrane (Mb) and soluble (Sol) fractions, while supernatant fractions (S/N) were collected prior to parasite lysis. HASPB levels were determined by immunoblotting with anti-HASPB 336 or anti-EF1-α as in (C).

This analysis also showed extracellular GFP staining on vesicular-like structures at the parasite surface (one example is shown in Fig. 4D, enlarged version Fig. S5B), suggesting that surface shedding of this reporter protein can occur *in vivo*. Using these high-resolution microscopy methods, no extracellular GFP was detected by either SIEM or TIEM in parasites expressing HASPB18–GFP G2A (data not shown). Overall, the new results generated with live GFP parasites here confirm that the first 18 residues of HASPB are sufficient to allow transport of this protein to the parasite plasma membrane and exposure on the surface, although this occurs in only a minority of cells in these experiments (as compared with the 30% of HASPB18–GFP accessible to biotinylation in Denny *et al*. (2000). However, the parasites used in these experiments were ‘late passage’ (i.e. had been passaged > 10 times in culture post-cycling through a susceptible BALB/c mouse). While it is known that endogenous HASPB is expressed at a lower level in late passage cells (see Fig. 5), it is possible that HASPB18–GFP levels are also reduced in these parasites, despite expression of the transgene from a non-regulated construct.

### HASPB surface exposure correlates with metacyclogenesis

To confirm that full-length endogenous *L. major* HASPB (rather than HASPB18–GFP) is also surface-exposed in differentiating parasites, flow cytometry was again used to identify live non-permeabilized parasites (selected by their negative staining with Sulfo-NHS-AMCA) on the basis of their surface exposure of both HASPB (recognized by the polyclonal antibody 336; Flinn *et al*., 1994) and the metacyclic-specific form of the parasite surface glycoconjugate, lipophosphoglycan (LPG; recognized by the mouse monoclonal, 3F12; Sacks and da Silva, 1987). Analyses were carried out over a time-course, correlating with parasite growth from early (2 days) through to late log phase (5 days) and into stationary phase (7 days), when metacyclogenesis takes place and metacyclic LPG is expressed. Both early and late passage parasites were used with the differences in HASPB expression levels between isolates confirmed by immunoblotting (Fig. 5C). This analysis showed a sevenfold increase in expression from early log to stationary phase in the early passage cells (similar to that seen in Sadlova *et al*., 2010) while HASPB expression is fivefold lower in late passage stationary-phase parasites.

The flow cytometry analysis in Fig. 5 shows that HASPB surface expression increases from 3% at Day 2 to at least 24% at Day 7 in early passage *L. major* with extracellular 3F12-expressing cells also staining with HASPB, confirming this double positive population as metacyclic parasites (Fig. 5A). Thus surface exposure of full-length HASPB correlates with entry into the mammalian-infective phase of the extracellular parasite growth cycle. By comparison, extracellular expression of HASPB in late passage *L. major* increases from 3% to only 10% from Day 2 to 7 (Fig. 5B) and, while all parasites displaying surface HASPB at Day 7 were identified as metacyclic by their surface expression of the 3F12 epitope, 28% of these parasites did not express surface HASPB; therefore, surface expression of HASPB is not required for metacyclogenesis *in vitro*, even though HASPB expression has been shown to be essential for the generation of metacyclic stages in the sand fly vector (Sadlova *et al*., 2010). This uncoupling of metacyclogenesis and HASPB expression suggests that the biochemical changes associated with LPG modification are not sufficient for the production of vector-transmissible parasites. Overall, the analysis in Fig. 5 demonstrates that cultured *L. major* metacyclics lose their ability to translocate HASPB across the plasma membrane in late passage parasites that have lowered infectivity for susceptible host strains.

Confocal microscopic analysis of early passage *L. major* wild-type metacyclics confirmed surface exposure of full-length HASPB across the entire cell body and flagellum (Fig. 5D) in a punctate pattern. Visualization of the closely associated 4′,6′-diamidino-2-phenylindole (DAPI)-stained kinetoplast and nucleus in this Sulfo-NHS-AMCA stained cell confirmed its status as a live metacyclic parasite, with extracellular HASPB and metacyclic LPG partially colocalizing on its surface and flagellum.

SIEM and TIEM analysis of early passage wild-type *L. major* parasites was carried out following live labelling with anti-HASPB 336 and secondary detection with a 10 nm gold-conjugated antibody. Surface HASPB was detected as clusters of 10 nm gold on the parasite body and flagellum by SIEM (Fig. 5E, enlarged version Fig. S5C), correlating with the punctate staining pattern observed by confocal analysis. Using TIEM, vesicles were detected at or leaving the cell surface and some of these carried surface gold particles, indicative of HASPB labelling, suggesting that HASPB translocated across the plasma membrane can be shed from the surface of metacyclic parasites (Fig. 5F, enlarged version Fig. S5D). All surface vesicles stained with 10 nm gold were 50–100 nm in diameter. Confocal microscopy, SIEM and TIEM were also carried out on late passage wild-type parasites, together with *L. major* mutant lines either deleted for or complemented with the LmcDNA16 locus encoding the HASP and SHERP genes (McKean *et al*., 2001; Sadlova *et al*., 2010); these are the cDNA16 KO and Kin lines respectively (as described in Table S1B). While surface labelling was visible in late passage wild-type cells, signal was more difficult to visualize at higher resolution in these low HASPB-expressing cells (Fig. S3A–C); as expected HASPB was not detected in the cDNA16 KO line (Fig. S3D–F) but cDNA16 Kin parasites displayed the wild-type phenotype (Fig. S3D, G and H).

### HASPB is shed from metacyclic parasites upon entry into host cell macrophages

To investigate whether HASPB is shed from the surface of metacyclics, cell fractionation was performed on Day 7 stationary phase early passage wild-type *L. major*, together with the mutant lines described above. Soluble and membrane fractions, plus culture supernatant samples collected after parasite removal by centrifugation, were immunoblotted with anti-HASPB 336 (Fig. 5G). As previously shown (Sadlova *et al*., 2010), HASPB expression is absent in the null (cDNA16 KO) parasites but restored in the complemented (Kin) parasites, in which it is differentially expressed although to a level ∼ 30% of wild type (Fig. 5G). Cell fractionation of metacyclics from all three lines demonstrated the presence of HASPB in both soluble and membrane fractions of the wild-type and complemented parasites, as expected for a dual acylated but not membrane-spanning protein. In addition, however, HASPB was also present in the supernatant fractions of both wild-type and complemented cells, strongly suggesting that HASPB can be released from the surface of metacyclic parasites, at least in culture (Fig. 5G). To confirm that the HASPB present in the supernatant fractions did not originate from dead cells, detection of the soluble cytosolic protein EF1-α was used as a control. This was detected in the soluble fractions of all three parasite lines, as expected, with some signal in the membrane fractions and detectable supernatant signal only present at low level in the wild-type parasite sample. From this analysis, we conclude that the HASPB detected in the supernatant fractions was not derived from dead parasites but rather, was released from live organisms.

**Fig 6:**
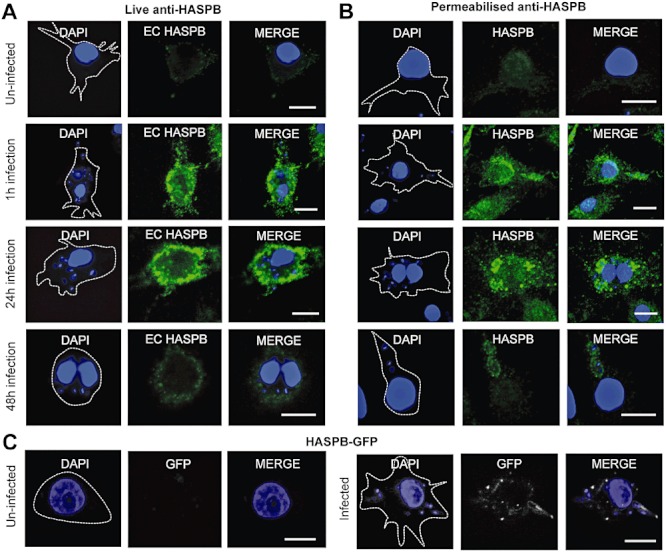
HASPB expression following macrophage uptake and amastigote differentiation. A and B. Bone marrow-derived mouse macrophages were incubated for 1, 24 and 48 h with stationary-phase wild-type *L. major* prior to labelling, either live (A) or after permeabilization (B), with rabbit anti-HASPB 336 and AlexaFluor-488 IgG to detect extracellular or total HASPB respectively by confocal microscopy with DAPI counter-staining. At 1 h of infection (second top panel), metacyclic parasites expressing HASPB are observed invading macrophages; at 24 h (third top panel), internal amastigote parasites are detected by DAPI and HASPB staining; at 48 h (bottom panel), amastigote infections are established. EC HASPB, extracellular HASPB. Size bar, 10 µm. C. Mouse macrophages were incubated for 24 h with stationary-phase late passage *L.major* transfected with full-length HASPB–GFP. Cells were fixed and DAPI counter-stained. White false-colour imagery was used to enhance visualization of GFP in these confocal images.

To determine the fate of surface HASPB during parasite phagocytosis, confocal microscopy was used to analyse bone marrow-derived mouse macrophages infected with early passage *L. major* wild-type metacyclic parasites over a 48 h time-course, prior to live staining with anti-HASPB336 and detection by AlexaFluor-488-conjugated secondary antibody. As metacyclic parasites attach to and enter the host macrophages after 1 h of incubation, HASPB is released from the parasite surface and shed onto the macrophage surface (Fig. 6A and B second panel). At 24 h of infection, phagocytosed parasites within macrophages are clearly visible by DAPI staining (Fig. 6A and B third panel). These internal parasites have a round body (Fig. 6B third panel) indicating differentiation to the amastigote stage. At this time, HASPB is detected on the surface of the macrophage (Fig. 6A third panel) and on the plasma membrane of internal parasites (Fig. 6B third panel). However, once amastigote infection is fully established at 48 h, surface HASPB is only weakly detectable (Fig. 6A bottom panel). In permeabilized infected macrophages HASPB is weakly detected in a punctate pattern on the amastigote plasma membrane at 48 h infection (Fig. 6B bottom panel). Less HASPB is detected on the macrophage surface after permeabilization, possibly due to plasma membrane disruption (Fig. 6B). These results suggest that HASPB is initially expressed in intracellular amastigotes but is downregulated once the parasites have undergone division within the parasitophorous vacuole. To address the possibility that the macrophage HASPB surface staining patterns observed were due to antibody capping, surface HASPB was detected using an alternative method without antibody staining. RAW mouse macrophages were incubated for 24 h with late passage metacyclic *L. major* episomally expressing full-length HASPB–GFP (Fig. 6C right panel). The surface distribution of HASPB–GFP on the macrophage following phagocytosis, although less easy to detect using these late passage parasites, confirms the observations made with antibodies, suggesting that HASPB may play a role in host/parasite interactions at this point of the infection cycle.

Confocal microscopy was also performed on RAW mouse macrophages which although less phagocytic than bone marrow-derived macrophages in our hands, establish strong amastigote infections by 48 h. Following infection with the cDNA16 KO and Kin parasite lines, no HASPB staining was detected in live or permeabilized macrophages infected with cDNA16 KO as expected (Fig. S4A), while cDNA16 Kin-infected macrophages displayed surface HASPB staining in cells with low parasite numbers but not in macrophages with high parasite numbers. These observations correlate with the more precisely timed wild-type infection data (Fig. 6). HASPB was also detected on the amastigote plasma membrane in permeabilized macrophages infected with the cDNA16Kin line (Fig. S4B), correlating with the wild-type phenotype seen in Fig. 6.

**Fig 7:**
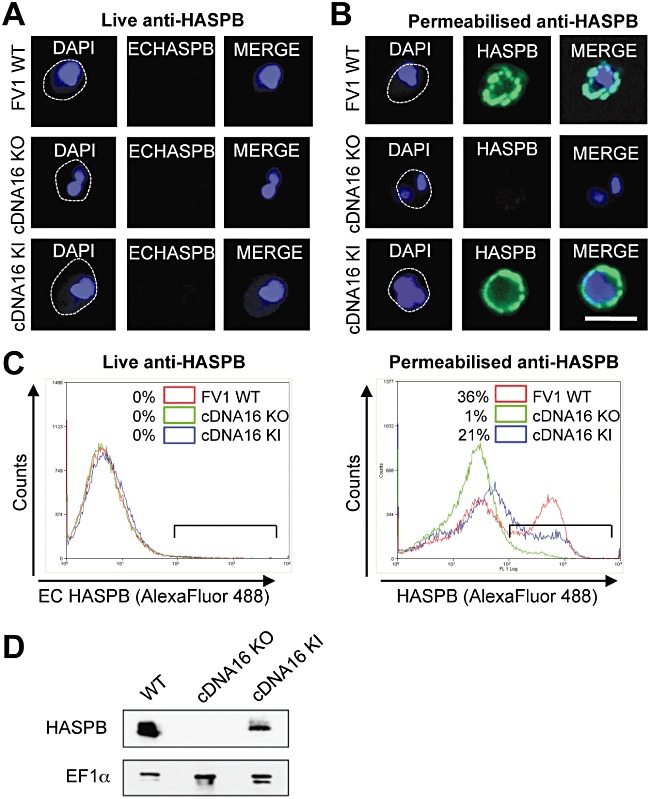
HASPB expression and localization in isolated amastigotes of wild-type and mutant *L. major*. Amastigotes of wild-type, LmcDNA16KO and Kin parasites were harvested from mouse macrophages after 48 h incubation for analysis by confocal microscopy and flow cytometry. A. Live amastigotes were labelled with anti-HASPB and anti-rabbit IgG AlexaFluor-488 and visualized by confocal microscopy, with DAPI counterstaining. B. Amastigotes were fixed and permeabilized prior to labelling as described in (A). C. Flow cytometry analysis of amastigotes labelled live as in (A) or after fixing as in (B). D. HASPB expression in the parasite lines analysed in (A)–(C), detected by immunoblotting as described in Fig. 5.

Confocal microscopy, flow cytometry and immunoblotting were also used to investigate HASPB localization and expression in isolated *L. major* amastigotes. FV1 wild-type, cDNA16 KO and Kin amastigotes were harvested from RAW mouse macrophages 48 h after infection and labelled live (Fig. 7A) and after permeabilization (Fig. 7B) with anti-HASPB 336, followed by detection with AlexaFluor-488-conjugated secondary antibody. No surface HASPB was observed on any of the three lines using these methods (Fig. 7A and C) but the protein was detected on the plasma membrane in 36% and 21% of permeabilized wild-type and cDNA16 Kin amastigotes respectively (Fig. 7B and C) compared with 1% background staining in the permeabilized cDNA16 KO amastigotes. These observations reflect the confocal data in Fig. 6B, which revealed differing HASPB expression in amastigotes within permeabilized macrophages depending on their stage of division in the host cell. Immunoblots of amastigote lysates showed HASPB expression in *L. major* wild type with partial complementation in the cDNA16 Kin parasites and no expression in the cDNA16 KO line as expected (Fig. 7D). Overall, these results demonstrate that HASPB is expressed in amastigotes, in which the protein is localized in a punctuate pattern at the plasma membrane in permeabilized cells. However, no surface-exposed HASPB could be detected using the methods described, either because the protein is not exposed at all on the amastigote surface or because it is rapidly cleared following surface exposure. Further, the results in Fig. 6 suggest that HASPB expression may be downregulated later during macrophage infection, following parasite replication, an observation that requires further analysis.

## Discussion

The *Leishmania* HASPB protein requires co- and post-translational acylation for localization to the plasma membrane and at this location, can be exposed on the external surface of the parasite, as detected by biotinylation (Denny *et al*., 2000). Here, we show that this pathway, originally identified using N-terminal HASPB18–GFP fusions expressed constitutively in non-infective procyclic cells, operates in metacyclic parasites expressing full-length endogenous HASPB. As shown recently, this is the only extracellular life cycle stage that expresses HASPB in the vector (Sadlova *et al*., 2010). Surface exposure of full-length HASPB in live metacyclics, confirmed using flow cytometry and imaging, is optimal in early passage parasites that also express metacyclic LPG (Fig. 5A). In comparison only a proportion of late passage metacyclics express both markers (Fig. 5B). When sorting early passage cells, two distinct populations of LPG/HASPB positive metacyclics are observed (Fig. 5A), perhaps indicative of the presence of a subpopulation of pre-apoptotic cells that have been shown to be essential for disease development following infection (van Zandbergen *et al*., 2006).

HASPB N-myristoylation is catalysed by myristoyl CoA: protein *N*-myristoyl transferase, a membrane-associated protein in *L. major*, *Drosophila* and mammalian cells (McIlhinney and McGlone, 1996; Ntwasa *et al*., 1997; Raju *et al*., 1997; Price *et al*., 2003). This modification targets HASPB to the Golgi region for subsequent palmitoylation and trafficking to the plasma membrane. The palmitoyl transferase responsible for this modification, and its localization in the parasite, has not yet been identified, although 10 DHHC-CRD proteins predicted to have palmitoyl acyltransferase activity are encoded in the *L. major* genome (L.M. MacLean, unpublished) with the homologue of one of these being required for protein sorting to the flagellar membrane in *Trypanosoma brucei* (Emmer *et al*., 2009). Live cell imaging of HASPB18–GFP C3S fusion protein in early passage *L. major* metacyclics detected non-palmitoylated HASPB18–GFP at the Golgi as previously but also (using the lipophilic probe FM4-64 for colocalization) at the flagellar pocket, lysosome and on vesicles at the posterior end of the parasite (Fig. 1B). These acidic vesicles, not previously detected in fixed cells, were more abundant in metacyclic parasites and were identified as a subset of autophagosomes by colocalization with RFP-ATG8 (Fig. 1D). One interpretation of these data is that Golgi accumulation of non-palmitoylated HASPB18–GFP unable to traffic to the plasma membrane leads to sequestration in autophagosomes prior to degradation. However, autophagosomes have also been implicated in the trafficking pathway of Acb1, another unconventionally secreted protein in yeast (Duran *et al*., 2010; Manjithaya *et al*., 2010). Given that HASPB is only expressed in metacyclics in the vector (Sadlova *et al*., 2010), it is interesting to note that macroautophagy in *Leishmania* correlates with parasite metacyclogenesis. Further analysis will be required to determine if autophagosomes play a role in transport of dual acylated HASPB to the plasma membrane; in the experiments described here, the fully acylated protein was not detected in the acidic vesicles colocalizing with RFP-ATG8.

In mammalian cells, the Src-family tyrosine kinases, important in the regulation of signal transduction, also have N-terminal SH4 domains like HASPB and localize to the cytoplasmic face of the plasma membrane by virtue of lipid modifications. Palmitoylation status determines the localization and modulates the function of specific proteins in this class (Sato *et al*., 2009) with palmitate addition and removal a rapid process at the cytoplasmic leaflet of Golgi membranes (Rocks *et al*., 2010). In yeast, the Vac8 vacuolar protein, which shares high sequence identity with the HASPB N-terminal SH4 domain, is compromised in its functions in morphology and inheritance in the absence of N-terminal palmitoylation (Subramanian *et al*., 2006). In kinetoplastid parasites, other dual acylated proteins characterized to date include the PPEF phosphatase and SMP-1 proteins in *Leishmania* species (Tull *et al*., 2004; 2010; Mills *et al*., 2007) and the *Trypanosoma cruzi* phosphoinositide-specific phospholipase C and flagellar calcium-binding protein (Godsel and Engman, 1999; Wingard *et al*., 2008; de Paulo Martins *et al*., 2010). Truncated versions of *Leishmania* PPEF and SMP-1 fused with reporter proteins are targeted to the flagellum, suggesting that transport to the flagellar membrane is the default pathway for dual acylated proteins. However, both full-length HASPB and HASPB18–GFP localize to the flagellum and cell body, suggesting that additional signals in the HASPB N-terminal 18 amino acids are required for retention at the plasma membrane.

As the HASPB export pathway is conserved in higher eukaryotes, mammalian cell mutants expressing HASPB18–GFP (generated by random retroviral insertion mutagenesis) have been used to investigate the trafficking pathway (Stegmayer *et al*., 2005). This work has led to the identification of an additional post-translational modification in the HASPB SH4 domain (phosphorylation at threonine-6) that influences plasma membrane targeting in mammalian cells by an endosomal recycling mechanism not associated with reduced levels of N-myristoylation or palmitoylation (Tournaviti *et al*., 2009). The experiments described here (Fig. 2) provide no evidence for HASPB phosphorylation in *Leishmania* metacyclics. Although the T6ES3ES4E *L. major* mutant protein mislocalized to the cytosol, P^32^-orthophosphate labelling revealed no detectable radiolabelled HASPB while the triple mutation led to disruption of the N-myristoylation site (Fig. 2), reflecting the divergence in the enzyme recognition site between *Leishmania* and mammalian cells. These observations suggest that a phosphorylation-dependent endosomal recycling system does not play a role in the movement of native HASPB from the Golgi to the plasma membrane. Rather, an alternative mechanism seems likely. While attempts to screen *Leishmania* genetically for effectors in this process have been challenging, a recent human genome-wide RNAi screen for factors involved in SH4-dependent protein targeting may be informative. Using SH4-HASPB–GFP and a mammalian SRC kinase YES-1 reporter (SH4-YES1-mCherry), 13 gene products were identified that may function primarily in HASPB targeting and a further 23 that are required for both HASPB and YES1 targeting (Ritzerfeld *et al*., 2011). Those with high scores in both HASPB and YES1 intracellular trafficking included COPB1 (a subunit of the coatomer complex), protein kinase C, the membrane receptors ITGAV (integrin alpha vitronectin) and ITGB1 (integrin beta 1), and PRP8 (pre-mRNA processing factor 8 homologue). Interestingly, downregulation of coatomer subunits COPB1 (β-COP) and ARCN1 (δ-COP) had a more marked effect on HASPB plasma membrane targeting. In mammalian cells, cytosolic vesicular coat proteins such as those of the COPI coatomer complex concentrate and package cargo molecules into vesicles to mediate efficient transport between intracellular compartments (Pucadyil and Schmid, 2009). Activated GTPases (e.g. Arf, Sar1 and dynamin) are core components of this coated vesicle machinery. Recent evidence shows that the COPI complex initially drives the formation of Golgi buds with subsequent lipid enzymatic activities determining vesicle fission events resulting in either retrograde or anterograde intra-Golgi transport and Golgi ribbon formation (Yang *et al*., 2011). While mechanistic understanding of these processes in kinetoplastid species is not yet well advanced, it is known that there is significant streamlining of the early secretory pathway in *T. brucei* (Sevova and Bangs, 2009). Thus, while studying human orthologues may provide mechanistic insight, parasite-specific features are more likely to be important in understanding non-classical secretion routes in *Leishmania* and other pathogens.

To investigate HASPB movement at the cell body membrane and flagellum of the highly motile *Leishmania* metacyclic parasite, we used FRAP analysis of live immobilized HASPB18–GFP *L. major*. This demonstrated that HASPB is bidirectionally mobile within the inner leaflet of the cell body membrane and within the flagellum (Fig. 3). Eukaryotic cilia and flagella share the same basic structure consisting of axonemal microtubules (9 + 2 or 9 + 0 doublet organization) extending from a basal body covered by membrane. The ciliary membrane has a distinct protein and high lipid composition compared with the periciliary and cell body membrane (Tetley *et al*., 1986; Tyler *et al*., 2009) and is separated from them by transition fibres. During cilium biogenesis, specific proteins are trafficked to and from the tip by the intraflagellar transport (IFT) machinery. In *T. brucei*, bidirectional GFP::IFT52 transport occurs rapidly at > 3 µm s^−1^ (Absalon *et al*., 2008), so it is possible that HASPB18–GFP is trafficked to and from the flagellar tip by the IFT machinery.

HASPB also moves back and forth between the cell body membrane and flagellar pocket but there is no detectable movement (during the time-course of these experiments) from the pocket or cell body membrane to the flagellum or vice versa, suggesting that HASPB movement to the flagellum is restricted. This observation was further supported by bleaching the entire cell body, resulting in swift recovery of fluorescence to the portion of the flagellum inserted into the pocket but not to the cell body membrane. These observations could be indicative of the presence of a physical ciliary gate and/or molecular diffusion barrier at the base of the flagellum that restricts movement of flagellar proteins to the cell body membrane (a concept recently reviewed in Nachury *et al*., 2010). There is some speculation about the existence of such a diffusion barrier at the base of cilia; however, recent reports suggest that septin2 is part of a diffusion barrier at the base of the ciliary membrane (Hu *et al*., 2010) and that plasma membrane proteins are anchored to the cortical actin cytoskeleton to restrict their movement to primary cilia (Breslow and Nachury, 2011). Our data suggest that protein transport from the flagellum to the cell body membrane is physically restricted in kinetoplastid parasites, although the mechanism involved remains to be elucidated.

Once exposed on the external face of the metacyclic plasma membrane, HASPB can be shed into the culture supernatant (Fig. 5G) and this shedding is associated with vesicles that are similar in size to the recently identified *L. donovani* exosomes (Silverman *et al*., 2010a,b). It is unclear whether HASPB is localized within these vesicles or is associated with the vesicular surface, however, given the presence of the dual acylated protein on both sides of the lipid bilayer. In the absence of further characterization of these structures, this remains an open question.

HASPB is also detected on the surface of macrophages during and immediately after phagocytosis (Fig. 6), where it shows a spreading punctuate pattern, presumably due to movement within the external lipid bilayer. Interestingly, surface spreading between cells was also observed in CHO mutant cell lines expressing HASPB–GFP (Stegmayer *et al*., 2005). In *Leishmania*, LPG epitopes have also been detected on the macrophage surface (Tolson *et al*., 1990), indicative of glycoconjugate release either during or after phagocytosis. Conversely, we have been unable to detect externally exposed HASPB on intracellular amastigotes within the macrophage parasitophorous vacuole, despite expression of this protein at increased level in this life cycle stage (this article; Price *et al*., 2003).

In conclusion, this study further elucidates the route taken by HASPB from its site of synthesis in the cytoplasm of metacyclic parasites to its surface display and shedding on to host macrophages. Conversely, in amastigotes, HASPB is expressed but not detectable on the cell surface of these intracellular parasites. It remains to be determined why this unusual protein avoids transport through the conventional secretory pathway of the parasite on its route to the surface and how and when it is released from metacyclics *in vivo*: in the sand fly prior to transmission or following inoculation into the host? Similarly, is HASPB in amastigotes exposed and removed from the parasite surface or retained within the cell? Further analysis, including vector transmission studies using *Leishmania* mutants deleted for the HASPB gene, should be informative in determining the function of this unusual protein in the *Leishmania* life cycle.

## Experimental procedures

### Leishmania culture

*Leishmania major* wild-type parasites (MHOM/IL/81/Friedlin, FV1 strain) were maintained at 26°C *in vitro*, inoculated into culture medium at 10^5^ ml^−1^ and grown from early logarithmic procyclic parasites (Day 2) to stationary-phase metacyclic parasites (Day 7) as previously described (Flinn *et al*., 1994). All parasites categorized as early passage cells were ≤ p6 while late passage cells were ≥ p10 relative to the time of first extraction from the lymph nodes of BALB/c mice.

**Transfected parasite lines.** The homozygous null (knockout, KO) *L. major* line, ΔcDNA16::HYG/ΔcDNA16::PAC, deleted for the diploid LmcDNA16 locus encoding the SHERP and HASP genes (McKean *et al*., 2001), and a complemented line (knock-in, Kin; ΔcDNA16::HYG/ΔcDNA16::PAC/ΔPAC::cDNA16) with a single copy of the locus inserted into its original genomic location on chromosome 23 by homologous recombination (Sadlova *et al*., 2010) were maintained under appropriate drug selection. *L. major* parasites expressing the N-terminal 18 amino acids of HASPB as a C-terminal fusion with GFP (HASPB18–GFP) on the pX NEO plasmid, the myristoylation-minus mutation of this transgene (HASPB18–GFP G2A) or the palmitoylation mutant of this transgene (HASPB18–GFP C3S) were grown in media supplemented with 100–500 ng ml^−1^ neomycin as described (Denny *et al*., 2000). Early passage HASPB18–GFP and HASPB18–GFP C3S were transfected with N-terminal RFP-tagged ATG8 [subcloned from ATG8-GFP into pNUS-HnRFP (Besteiro *et al*., 2006; Williams *et al*., 2006), the gift of Jeremy Mottram, University of Glasgow] and grown in medium additionally supplemented with 10 µg ml^−1^ blasticidin.

The complete HASPB ORF was amplified from the *L. major* cDNA16 Kin DNA construct described above, using proofreading DNA polymerase (KOD, Merck, UK) and primers HASPBFLf and HASPBFLr (listed in Table S2), then subsequently cloned into the CT-GFP TOPO vector (Invitrogen), generating a C-terminal GFP fusion. HASPB–GFP was amplified from this construct and cloned into pX NEO, prior to transfection of early passage *L. major* FV1 using the AMAXA system and selection/characterization of clones as previously described (Price *et al*., 2003; Brannigan *et al*., 2010).

Using the HASPB18–GFP pXNEO construct described above, *L. major* lines were generated with single (T6E, S3E, S4E) or triple (T6ES3ES4E) threonine and/or serine to glutamate mutations at potential phosphorylation sites within the N-terminal HASPB-18 amino acids, using site-directed mutagenesis (GeneTailor Site-Directed Mutagenesis System, Invitrogen). A triple alanine mutation was also generated (T6AS3AS4A). Specific forward site-directed mutagenesis primers and generic single and triple mutant reverse primers (HASPB18r and T6AES3S4r) used for PCR amplification of these constructs are listed in Table S2 with sequence analysis primer GFPr. All plasmids generated were used for parasite transfection as described above, with transfected lines maintained in media supplemented with neomycin.

### *In vitro* macrophage infection and amastigote isolation

Bone marrow derived mouse macrophages and RAW 264.7 mouse macrophages were maintained at 37°C *in vitro* as previously described (Depledge *et al*., 2009). For microscopic analysis, 5 × 10^5^ macrophages were seeded at 2 ml per well and allowed to adhere on to coverslips for 2 h at 37°C. After removal of media, parasites were added at 5 × 10^6^ per well and incubated for 2 h at 34°C, before washing macrophages twice with Dulbecco's modified Eagle's medium (DMEM), replacement with 2 ml per well fresh complete DMEM and further incubation for 1–48 h at 34°C. To harvest amastigotes, 100 ml of confluent RAW 264.7 macrophages were incubated with 100 ml of metacyclic-rich parasites (∼ 2 × 10^9^ cells) for 2 h at 34°C, then washed twice with DMEM. Infected macrophages were then incubated for a further 24–48 h at 34°C in 100 ml of fresh complete DMEM before harvesting, using 0.05% saponin and a single density isotonic Percoll gradient as previously described (Depledge *et al*., 2009).

### Confocal microscopy

**Live cell imaging.** Early passage *L. major* HASPB18–GFP, C3S mutants and full-length HASPB–GFP parasites (all at 10^7^) were collected by centrifugation (800 *g* for 10 min), washed in PBS, resuspended in 5 µl of PBS and treated with 40 µM lipophilic dye FM4-64 or 50 nm Lysotracker Red DND-99 (Invitrogen) prior to immobilization in 250 µl of cold PBS-primed CyGEL (Biostatus) for live cell imaging as described (Price *et al*., 2010). Parasites were imaged after 90 min using a Zeiss LSM510 meta confocal microscope with a Plan-Apochromat 63×/1.4 Oil DIC I objective. FM4-64 and Lysotracker RED were excited at 543 nm and emission collected using a 560 LP filter.

FRAP analysis was performed on live mid- to late-log-phase parasites immobilized in PBS-primed CyGEL. GFP transgene expression was visualized by confocal microscopy, using the 488 nm laser for excitation with emission collected through a 505 LP filter. ROIs of a fixed dimension were chosen and the laser scanned in the selected ROI with 100 iterations at an elevated laser power. Pre- and post-bleach images were collected as part of a time series up to 156 s (with a further 37 min time point in Fig. 3Aiv). Analysis was performed using SigmaPlot11 and data fitted according to a single exponential.

**Antibody labelling and imaging.** Labelling was performed on live parasites and mouse macrophages (to detect surface proteins, using methods designed to minimize antibody capping, see below) and on permeabilized cells (to detect total protein localization). Prior to live cell staining, the amine-reactive fluorophore Sulfo-NHS-AMCA (Pierce) was used to confirm cell viability; dead cells stained with this reagent emit a strong blue fluorescence (Engstler *et al*., 2007; Depledge *et al*., 2010). A total of 2 × 10^7^ parasites were collected by centrifugation at 800 *g* for 10 min, washed and incubated with Sulfo-NHS-AMCA (1 mM) on ice for 10 min, before termination of the reaction by addition of 10 mM Tris pH 8.5 (Grunfelder *et al*., 2003). Cells were then washed three times with cold 1% fatty acid-free BSA blocking solution (BB International) and resuspended in 100 µl of blocking solution for 20 min. To detect extracellular (EC) HASPB and lipophosphoglycan (LPG), parasites were labelled with rabbit anti-HASPB336 (1:300) (Flinn *et al*., 1994) and/or mouse 3F12 (undiluted) (Sacks and da Silva, 1987) for 30 min at 20°C, then fixed in 4% paraformaldehyde (PFA) before secondary detection with AlexaFluor-488-conjugated goat anti-rabbit IgG and AlexaFluor-647-conjugated goat anti-mouse IgG respectively (1:250 in blocking solution, Invitrogen). Extracellular GFP was also detected on HASPB18–GFP parasites by incubation with mouse anti-GFP (1:200; Invitrogen), using the live cell labelling and imaging methods described above.

**Fixed cell imaging.** *L. major* transgenic parasites at different stages were fixed in 4% PFA, washed in PBS, allowed to adhere to polylysine slides (Sigma) for 20 min and mounted with Vectashield containing DAPI (Vector Laboratories). Mouse macrophages were fixed as described above but first adhered to glass coverslips. Labelling was also carried out on permeabilized cells by the addition of 0.2% Triton X-100 (Sigma) in blocking solution for 10 min after PFA fixation. The same protocol was followed for Golgi colocalization studies using rabbit anti-YPT/Rab 1 (1:100; the gift of Emanuela Handman, Melbourne, Australia).

### Flow cytometry

Parasite surface and total HASPB, LPG and GFP were quantified by flow cytometry. Parasites were labelled live and permeabilized according to the confocal microscopy protocol above. A proportion of each sample was removed before slide preparation, analysed on a Dako CyAn ADP and data evaluated using Summit 4.3 Software.

### SIEM and TIEM

**SIEM.** Stationary-phase parasites (10^7^) were adhered to coverslips coated with 100 ng ml^−1^ poly-l-lysine (Sigma) for 25 min before labelling. Parasites were labelled live with rabbit anti-HASPB336 (1:300) at 20°C for 15 min, fixed in 4% PFA (as previously described) and washed three times in 20 mM glycine pH 7.4. Surface HASPB was detected by goat anti-rabbit 10 nm gold conjugated IgG (1:10) (BB International) and cells were post-fixed in 2.5% gluteraldehyde, prior to washing in phosphate buffer, dehydration through an ethanol series (25%, 50%, 70%, 90%, 100%) and storage in hexamethyldisilazane (HMDS) overnight. When dry, samples were mounted on stubs and carbon coated. Images were collected on a JSM-7500F (JEOL, Japan) scanning electron microscope.

**TIEM.** A total of 5 × 10^8^ stationary-phase parasites were labelled as described above. Cells were post-fixed in 2.5% glutaraldehyde, 4% formaldehyde and dehydrated in an acetone series (Price *et al*., 2003) before embedding in Spurr resin. Sections were cut using a Leica UCT ultramicrotome at 60 nm and counterstained with saturated uranyl acetate in 50% ethanol, Reynolds lead citrate for 5 min. Samples were examined using a Tecnai 12 BioTWIN (FEI) transmission electron microscope and images collected using a SIS Megaview III camera.

### Parasite metabolic labelling and immunoprecipitation

**^3^H myristate and palmitate.** Parasites were radiolabelled with fatty acids by coupling ^3^H myristate and ^3^H palmitate (Perkin Elmer) to BSA, followed by incubation with parasites starved of FCS. Briefly 3.7 MBq of each tritiated fatty acid was incubated for 1 h at 20°C (shaking) with 400 µl of M199 medium supplemented with 1.8% fatty acid-free BSA (BB International). A total of 2 × 10^9^ parasites were washed (3 × 10 min 800 *g*), and resuspended in 300 µl of M199 without FCS plus 1.8 mg ml^−1^ fatty acid-free BSA, and incubated for 60 min at 26°C. FCS starved parasites were incubated with ^3^H fatty acid-coupled BSA for 3 h at 26°C. Cells were washed in cold PBS and resuspended in 1 ml of cold lysis buffer {10 mM Tris pH 7.5, 1 mM EDTA, 50 mM NaCl, + protease inhibitors [4 mg ml^−1^ pepstatin A, phosphatase inhibitor cocktail II 1:700 (Calbiochem), 2 mM Na pyrophosphate, 2 mM beta-glycero-phosphate, 1 protease inhibitor tablet per 7 ml (Roche)]}, sonicated on ice for 3 × 10 s, detergents were then added (1% NP40, 0.5% deoxycholic acid, 0.1% SDS) and the lysates incubated on ice for 30 min. These were then centrifuged for 30 min 4°C at 13 000 r.p.m. and the soluble fraction (between the upper lipid film and membrane pellet) transferred to a fresh tube, labelled as total lysate.

**Immunoprecipitation with anti-GFP.** One hundred microlitres of protein A-coupled beads (Dynabeads, Invitrogen) were pre-washed in 500 µl of cold 0.1 M sodium phosphate buffer pH 8 (3× at 4°C) and resuspended in 90 µl of cold 0.1 M sodium phosphate buffer pH 8. After addition of 10 µl of rabbit anti-GFP antibody (Abcam ab290), the mix was rotated (10 min at 20°C) and the beads washed (3× in 500 µl of cold 0.1 M sodium phosphate buffer pH 8, 0.01% Tween). For cross-linking, 1 ml of 0.2 M triethanolamine pH 8.2 was added to the beads, followed by washing (2× in 500 µl of triethanolamine) and resuspension in 1 ml of 20 mM dimethyl pimelimidate·2HCl (DMP, Thermo Scientific) in 0.2 M triethanolamine pH 8.2 (prepared just before use). After rotation for 30 min at 20°C, the supernatant was removed and the beads resuspended in 1 ml of 50 mM Tris pH 7.5, prior to rotation for 15 min at 4°C. The samples were then washed (3× PBS pH 7.4) and resuspended in 100 µl of PBS pH 7.4. The soluble fraction of the labelled cells (total lysate, prepared as described above) was added to the antibody-coupled beads and the mixture left to rock slowly for 1 h at 4°C. The remaining lysate was then removed and kept as the ‘depleted’ sample. Beads were washed (3× in 1 ml of cold lysis buffer with fresh inhibitors and detergent) followed by three washes in ice-cold PBS to remove detergent. Thirty microlitres of SDS-PAGE buffer was added and GFP-immunoprecipitated products (GFP IP) eluted by heating for 5 min at 65°C to ice and collection of the supernatant after centrifugation.

Ten microlitres of all samples were separated by SDS-PAGE, the gels stained with Coomassie blue, washed in H_2_O for 1 h and incubated in EN^3^HANCE solution (Perkin Elmer) for 1 h. After further washing in H_2_O followed by incubation in 1% glycerol, 5% PEG 8000 for 1.5 h, gels were dried (2 h at 80°C) and exposed for autoradiography for 28 days (^3^H myristate-labelled) or 135 days (^3^H palmitate-labelled).

**^32^P orthophosphoric acid.** A total of 2 × 10^9^ metacyclic *L. major* were collected by centrifugation (800 *g*) and washed 3× in Krebs buffer without phosphate or FCS (120 mM NaCl, 1 mM KCl, 1.25 mM CaCl_2_, 1.2 mM MgSO_4_, 25 mM NaHCO_3_, 10 mM glucose, 5 mM Hepes pH 7.4). Cells were resuspended in 0.2 ml of 2× Krebs buffer without phosphate plus 0.2 mCi ^32^P orthophosphate (Perkin Elmer) and incubated at 26°C for 3 h. After washing the labelled parasites (3× in PBS at 4°C), cells were lysed and immunoprecipitations carried out as described above. All samples were analysed by SDS-PAGE, followed by Coomassie blue staining and autoradiography (using EN^3^HANCE solution and storage at −80°C).

### Cell fractionation and immunoblotting

*Leishmania major* parasite lines were grown for 24 h in Schneider's medium at pH 5.5 (Invitrogen) minus FCS. A total of 4 × 10^8^ parasites were collected from 100 ml of cultures by centrifugation at 800 *g* for 10 min at 4°C. One hundred millilitres of each supernatant was loaded onto successive spin columns (Vivaspin20, Sartorious 5 kD and Amicon Ultra 4, Millipore 5 kD) for concentration to a final volume of 200 µl by centrifugation at 3500 *g* at 4°C. An equal volume of 2× Laemmli buffer was added to produce the ‘supernatant fraction’. For the parasite fractions, cells were washed twice in 10 ml of cold PBS (800 *g* 10 min, 4°C) and resuspended in 400 µl of lysis buffer containing protease inhibitors (as described above) on ice. Cells were lysed by sonication on ice (4 × 10 s) and undisrupted cells removed by centrifugation at 100 *g* for 2 min at 4°C. Soluble and membrane fractions were separated by ultracentrifugation at 100 000 *g* for 1 h at 4°C. The resulting supernatant was removed and an equal volume of 2× Laemmli buffer added to produce the ‘soluble fraction’. The remaining membrane fraction was washed twice in cold PBS, resuspended in 400 µl of lysis buffer and an equal volume of 2× Laemmli buffer added, generating the ‘membrane fraction’.

For sucrose gradient fractionation of lysates from HASPB18–GFP and C3S/RFP-ATG8 *L. major*, 1 × 10^9^ PBS-washed cells were resuspended in 5 ml of hypotonic buffer (2 mM EGTA, 2 mM DTT, 0.23 mM leupeptin, 0.1 mM PMSF) and lysed by repeated (×15) expulsion through a 27-gauge needle. The lysate was made isotonic by addition of 4× assay buffer (50 mM Hepes-NaOH, 0.25 M sucrose, 1 mM ATP, 1 mM EGTA, 2 mM DTT, 0.2 mM leupeptin, 0.1 mM PMSF, pH 7.4) and centrifuged at 3000 *g* for 10 min. The supernatant was applied to two sucrose gradients, prepared by layering 0.8 ml fractions of 0.25–2.25 M sucrose (in 25 mM Hepes-NaOH, pH 7.4) over a cushion of 2.5 M sucrose in Ultraclear centrifuge tubes and pre-centrifugation at 218 000 *g* for 1 h at 4°C, using a SW41Ti rotor in a Beckman L60 Ultracentrifuge. After addition of the cell lysate, the tubes were centrifuged at 218 000 *g*, 4°C for 6 h and 0.5 ml fractions collected for analysis. Sucrose gradient fractions were concentrated fourfold and buffer exchanged into 25 mM Hepes-NaOH, pH 7.4 using Vivaspin 500 centrifugal concentrators (Vivascience).

**Immunoblots.** The equivalent of 5 × 10^6^ cells per fraction were separated by 10% SDS-PAGE (NuPage) and proteins transferred on to nitrocellulose membrane (Millipore). The resulting blots were probed with rabbit anti-HASPB336 (1:3000) or mouse anti-EF1-α (Millipore; 1:2000) and appropriate secondary antibodies. Immune complexes were detected using ECL reagents (Amersham Biosciences) and images analysed by densitometry using ImageJ software. The same immunoblotting procedure was used to detect GFP in transgenic parasite lysates, using rabbit polyclonal anti-GFP (Invitrogen, 1:2000) for detection. Sucrose gradient fractions were probed with rabbit anti-GFP, rabbit anti-RFP (1:1000 Abcam) and rabbit anti-GP63 (1:400; a gift from Robert MacMaster) as described above.

## Supporting information

Additional Supporting Information may be found in the online version of this article: **Fig. S1.** A. Live cell analysis of metacyclicparasites expressing the full-length HASPB–GFP fusion proteinand stained with Lysotracker RED DND-99. Size bar, 5 μm. B. Statistical analyses of HASPB18–GFP FRAP replicates.Analyses were repeated at least four times for each ROI in Fig.3Ai–iii. HASPB18–GFP C3S vesicle FRAP was repeatedtwice. Data are graphically represented as mean recovery times(*t*_1/2_) with SE bars. C. FV1 wild-type, HASPB18–GFP and G2A parasites were lysed,separated into membrane and cytosolic fractions and analysed forHASPB18–GFP expression by immunoblotting, usinganti-GFP. D. Confocal microscopic analysis of a metacyclic HASPB18–GFPG2A *L. major* labelled using the same protocol as in Fig. 4.Size bar, 5 μm.

**Fig. S2.** Protein loading comparison ofHASPB18–GFP and mutant reporter constructs analysed foracylation status in Fig. 2. A. Coomassie-stained gels of ^3^H-myristate-radiolabelledlysate and products immunoprecipitated with anti-GFP. B. Coomassie-stained gels of ^3^H-palmitate-radiolabelledlysate and products immunoprecipitated with anti-GFP.

**Fig. S3.** Microscopic analyses of HASPBlocalization in late passage FV1 *L. major* and LmcDNA16mutant metacyclic parasites A. Confocal microscopy of live anti-HASPB- and 3F12-labelled latepassage *L. major* (as described in Fig. 5D). Size bar, 5μm. B and C. (B) Scanning immunoelectron microscopy and (C)transmission immunoelectron microscopy of live anti-HASPB-labelledlate passage stationary-phase *L. major* detected with goatanti-rabbit IgG 10 nm gold. D. Confocal microscopy of cDNA16 KO (top panel) and Kin (lowerpanel) FVI *L. major*. Stationary-phase cells incubated livewith anti-3F12 and anti-HASPB (as described in Fig. 5D). Size bar,5 μm. E–H. (E and G) Scanning immunoelectron microscopy and (F andH) transmission immunoelectron microscopy of cDNA16 KO andcDNA16Kin *L. major* respectively labelled live withanti-HASPB (as described in Fig. 5). White arrows indicate gold particles in SEM image. The black box within the whole-cell image TIEM represents the area enlarged on the right; black arrows indicate labelled surface vesicles. F, flagellum; AE, anterior end.

**Fig. S4.** HASPB shedding during entry of cDNA16KO and Kin *L. major* parasites into macrophages and HASPBexpression in amastigotes within the parasitophorous vacuole.Confocal microscopy of mouse macrophages infected withstationary-phase (A) cDNA16 KO and (B) cDNA16 Kin *L. major*for 24 h and labelled live with rabbit anti-HASPB (top threepanels) and after permeabilization (lower three panels) to detectextracellular (EC) and total HASPB respectively. Early infectionwas defined as one parasite per vacuole and late infection wasdefined as multiple (2–10) parasites. Size bar, 10 μm.

**Fig. S5.** Enlarged versions of scanning and transmission immunoelectron micrographs in Fig. 4C and D and Fig. 5E and F.

**Table S1.** Cell lines used in this study. **Table S2.** Primers used in this study.

**Movie S1.** FRAP at the plasma membrane, *L.major* HASPB18-GFP.

**Movie S2.** FRAP at the flagellar pocket, *L.major* HASPB18-GFP.

**Movie S3.** FRAP at the flagellum, *L.major* HASPB18-GFP.

**Movie S4.** FRAP at the cell body, *L.major* HASPB18-GFP.

**Movie S5.** FRAP at intracellular vesicles, *L.major* HASPB18-GFP C3S.
