# Supplementary figures and images for: Trafficking and release of Leishmania metacyclic HASPB on macrophage invasion
Source: Cell Microbiol. 2012 Feb 24;14(5):740–61. doi: 10.1111/j.1462-5822.2012.01756.x (PMC3491706; doi:10.1111/j.1462-5822.2012.01756.x)

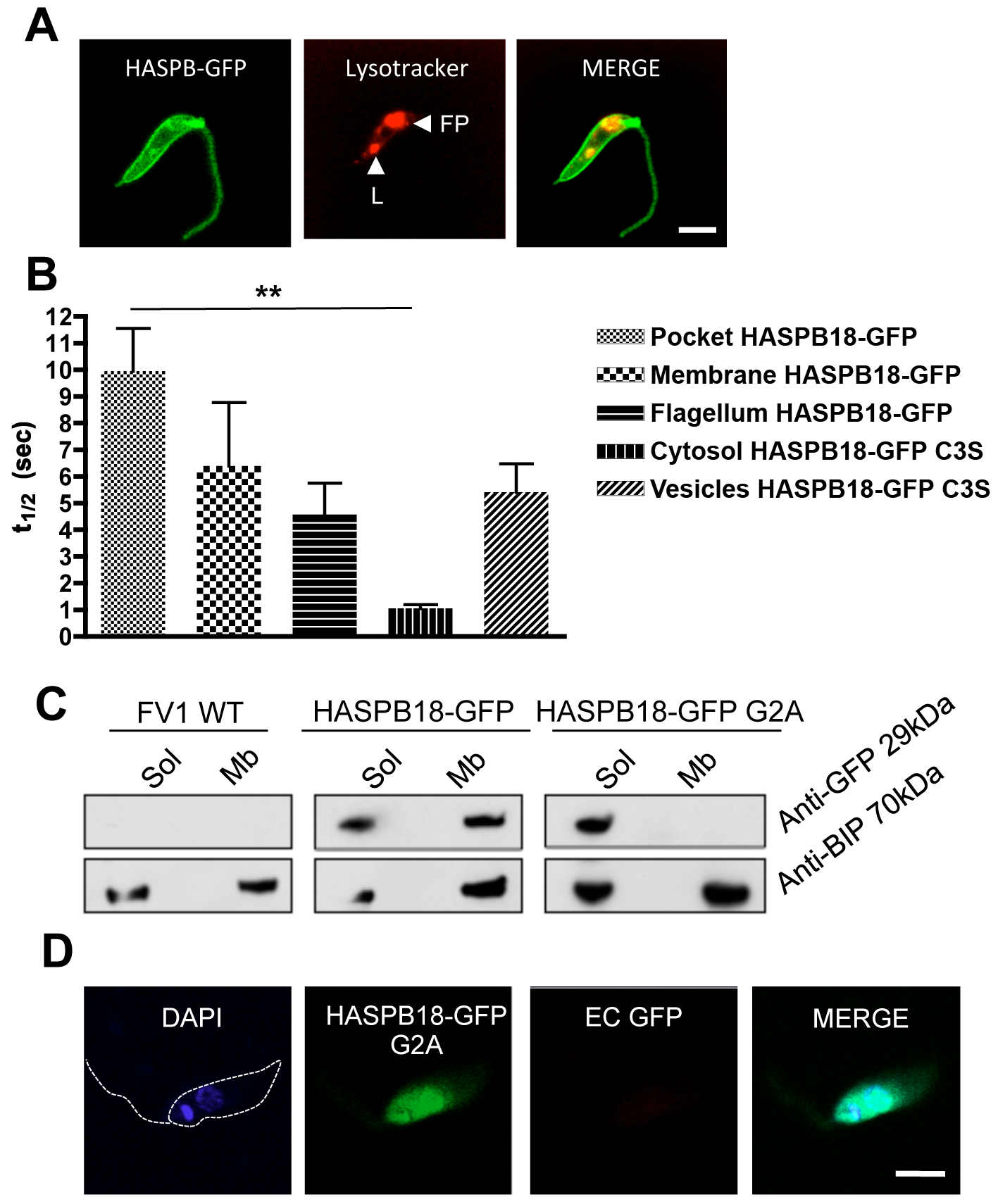

Supplement: Supplementary file 1 — Additional Supporting Information may be found in the online version of this article: Fig. S1. A. Live cell analysis of metacyclicparasites expressing the full-length HASPB–GFP fusion proteinand stained with Lysotracker RED DND-99. Size bar, 5 μm. B. Statistical analyses of HASPB18–GFP FRAP replicates.Analyses were repeated at least four times for each ROI in Fig.3Ai–iii. HASPB18–GFP C3S vesicle FRAP was repeatedtwice. Data are graphically represented as mean recovery times(t1/2) with SE bars. C. FV1 wild-type, HASPB18–GFP and G2A parasites were lysed,separated into membrane and cytosolic fractions and analysed forHASPB18–GFP expression by immunoblotting, usinganti-GFP. D. Confocal microscopic analysis of a metacyclic HASPB18–GFPG2A L. major labelled using the same protocol as in Fig. 4.Size bar, 5 μm. [file cmi0014-0740-SD1.tif]

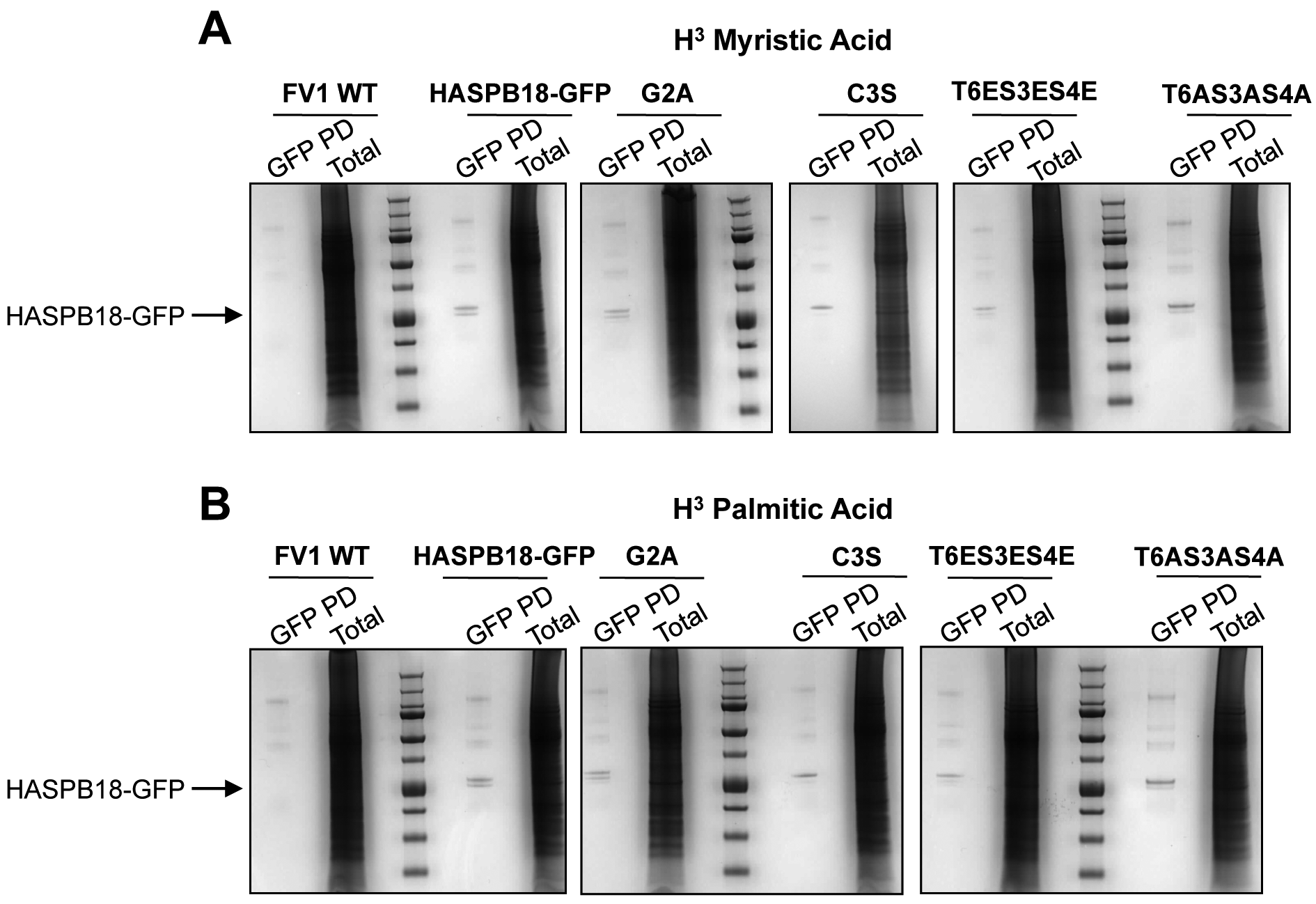

Supplement: Supplementary file 2 — Fig. S2. Protein loading comparison ofHASPB18–GFP and mutant reporter constructs analysed foracylation status in Fig. 2. A. Coomassie-stained gels of 3H-myristate-radiolabelledlysate and products immunoprecipitated with anti-GFP. B. Coomassie-stained gels of 3H-palmitate-radiolabelledlysate and products immunoprecipitated with anti-GFP. [file cmi0014-0740-SD2.tif]

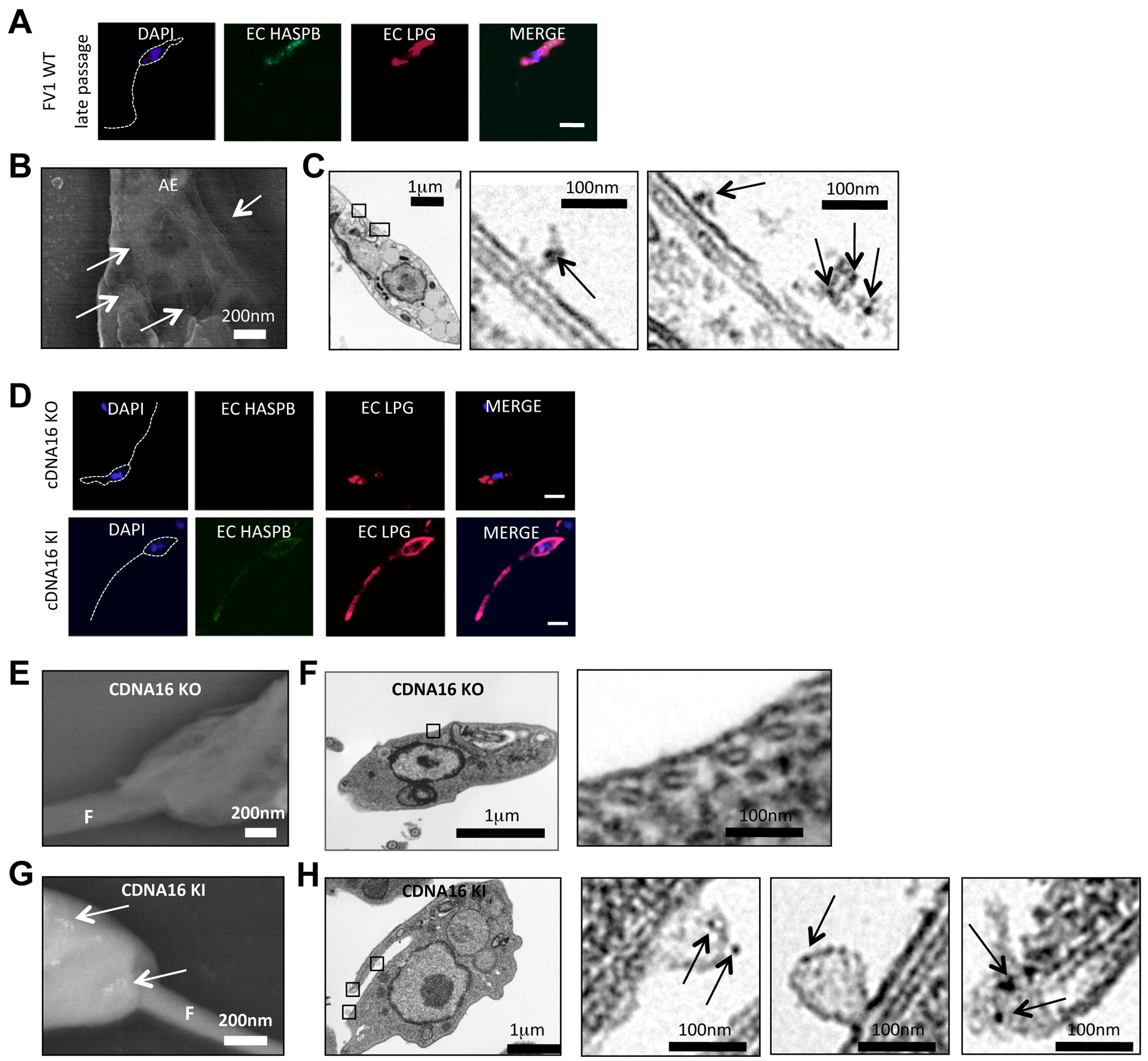

Supplement: Supplementary file 3 — Fig. S3. Microscopic analyses of HASPBlocalization in late passage FV1 L. major and LmcDNA16mutant metacyclic parasites A. Confocal microscopy of live anti-HASPB- and 3F12-labelled latepassage L. major (as described in Fig. 5D). Size bar, 5μm. B and C. (B) Scanning immunoelectron microscopy and (C)transmission immunoelectron microscopy of live anti-HASPB-labelledlate passage stationary-phase L. major detected with goatanti-rabbit IgG 10 nm gold. D. Confocal microscopy of cDNA16 KO (top panel) and Kin (lowerpanel) FVI L. major. Stationary-phase cells incubated livewith anti-3F12 and anti-HASPB (as described in Fig. 5D). Size bar,5 μm. E–H. (E and G) Scanning immunoelectron microscopy and (F andH) transmission immunoelectron microscopy of cDNA16 KO andcDNA16Kin L. major respectively labelled live withanti-HASPB (as described in Fig. 5). White arrows indicate gold particles in SEM image. The black box within the whole-cell image TIEM represents the area enlarged on the right; black arrows indicate labelled surface vesicles. F, flagellum; AE, anterior end. [file cmi0014-0740-SD3.tif]

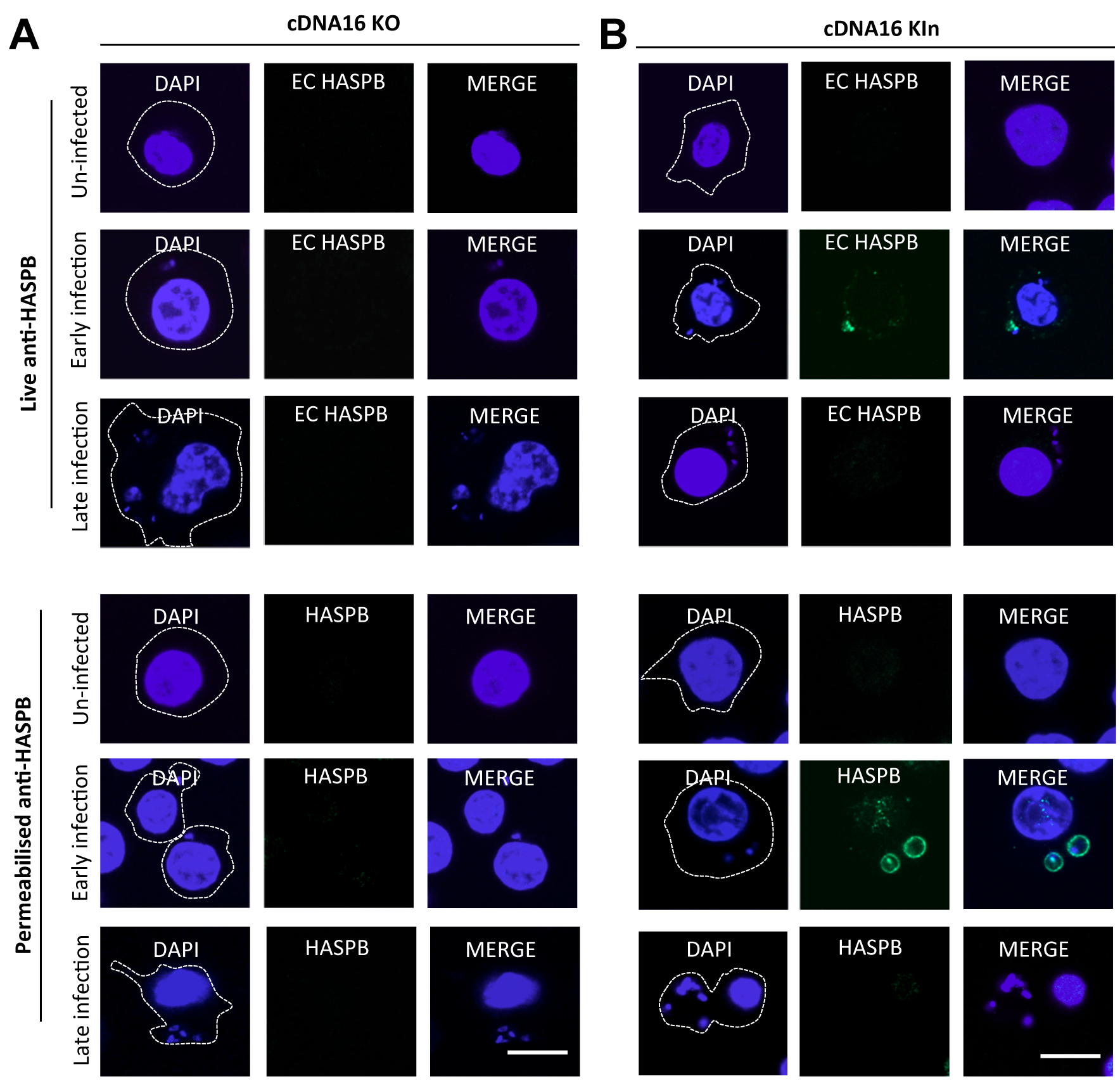

Supplement: Supplementary file 4 — Fig. S4. HASPB shedding during entry of cDNA16KO and Kin L. major parasites into macrophages and HASPBexpression in amastigotes within the parasitophorous vacuole.Confocal microscopy of mouse macrophages infected withstationary-phase (A) cDNA16 KO and (B) cDNA16 Kin L. majorfor 24 h and labelled live with rabbit anti-HASPB (top threepanels) and after permeabilization (lower three panels) to detectextracellular (EC) and total HASPB respectively. Early infectionwas defined as one parasite per vacuole and late infection wasdefined as multiple (2–10) parasites. Size bar, 10 μm. [file cmi0014-0740-SD4.tif]

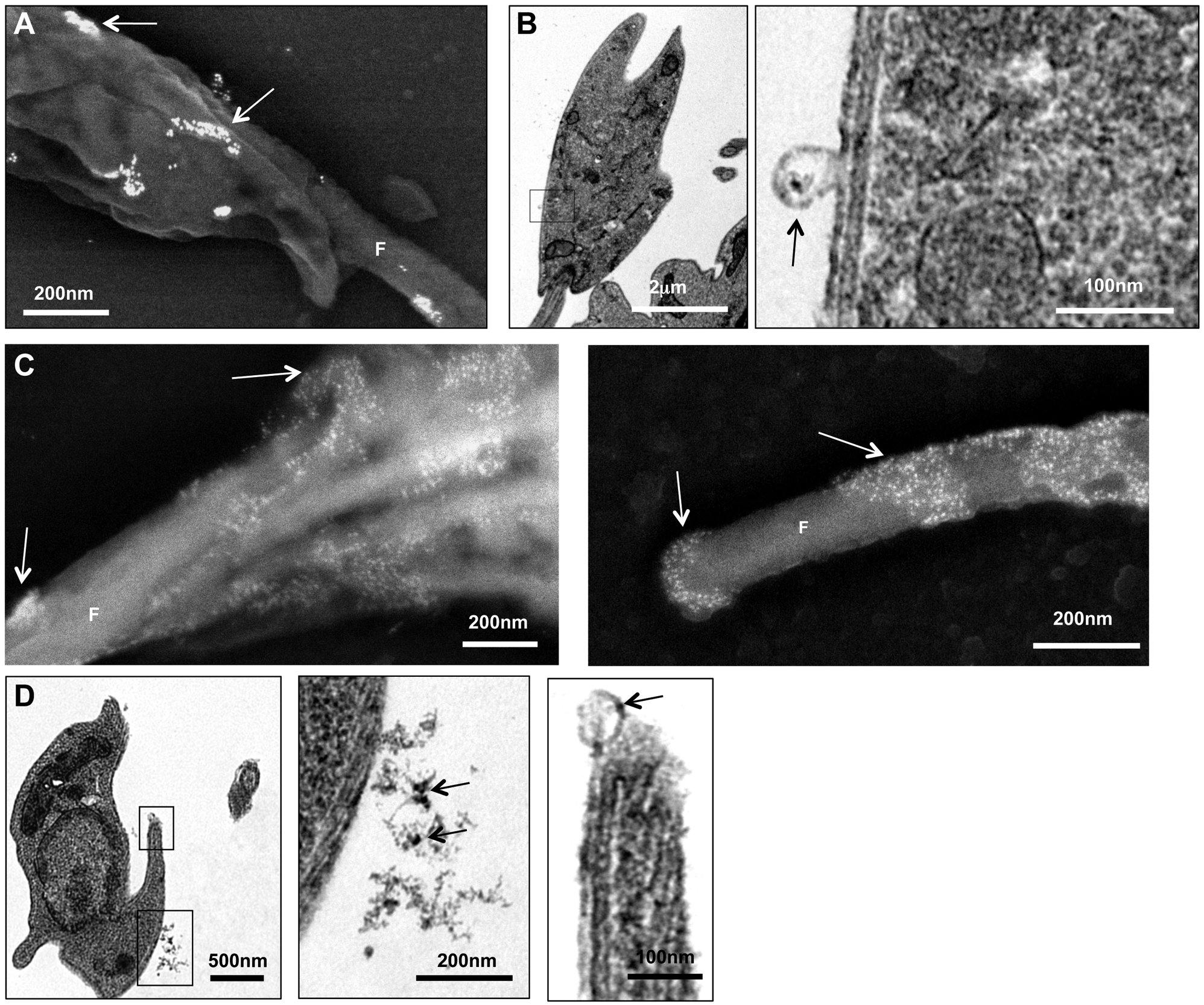

Supplement: Supplementary file 5 — Fig. S5. Enlarged versions of scanning and transmission immunoelectron micrographs in Fig. 4C and D and Fig. 5E and F. [file cmi0014-0740-SD5.tif]
