# Supplementary material for: Trafficking and release of Leishmania metacyclic HASPB on macrophage invasion
Source: Cell Microbiol. 2012 Feb 24;14(5):740–61. doi: 10.1111/j.1462-5822.2012.01756.x (PMC3491706; doi:10.1111/j.1462-5822.2012.01756.x)
Supplement: Supplementary file 6 — Table S1. Cell lines used in this study. Table S2. Primers used in this study. [file cmi0014-0740-SD6.doc]

**Table S1**. Cell lines used in this study

A.

| **Transgene** | **N-terminal sequence** | **Localisation** |
| --- | --- | --- |
| HASPB18-GFP | MGSSCTKDSAKEPQKSAD | Plasma membrane |
| HASPB18-GFP G2A | MASSCTKDSAKEPQKSAD | Cytosol |
| HASPB18-GFP C3S | MGSSSTKDSAKEPQKSAD | Golgi |
| HASPB-GFP | Full HASPB ORF | Plasma membrane |

B.

| **Cell line** | **Description** | **Genotype** |
| --- | --- | --- |
| cDNA16 KO | Homozygous null, deleted for diploid LmcDNA16 locus encoding SHERP, HASPA and HASPB genes | ΔcDNA16::HYG/ΔcDNA16::PAC |
| cDNA16 Kin | cDNA16 KO complemented with a single copy of the locus in its genomic location on chromosome 23 | ΔcDNA16::HYG/ΔcDNA16::PAC/ΔPAC::cDNA16 |

**Table S2**. Primers used in this study

| **Name** | **Sequence** |
| --- | --- |
| HASPBFLf | 5’-gatatgggaagctcttgcacgaa-3’ |
| HASPBFLr | 5’-cgttgccggcagcgtg-3’ |
| HASPB18T6Ef | 5’-cttatgggaagctcttgcgagaaggactccg-3’ |
| HASPB18S3Ef | 5’-tccccgggggatccatgggagagtcttgcacgaagga-3’ |
| HASPB18S4Ef | 5’-tccccgggggatccatgggaagcgagtgcacgaagga-3’ |
| HASPB18T6AS3AS4Af | 5’-tccccgggggatccatgggagccgcttgcgcgaagga-3’ |
| HASPB18T6ES3ES4Ef | 5’-tccccgggggatccatgggagaggagtgcgagaagga-3’ |
| HASPB18r | 5’-gcaagagcttcccataagcttgtgaattttac-3’ |
| T6AES3S4r | 5’-ggagggggacaggggccccctaggtaccct-3’ |
| GFPr | 5’-gctacatacggaaagcttaccc-3’ |
